# Decoding Past Microbial Communities Shifts Induced by Natural and Anthropogenic Disturbance Events Through Extracellular DNA

**DOI:** 10.1111/mec.70078

**Published:** 2025-08-22

**Authors:** S. Varrella, M. Tangherlini, C. Corinaldesi, L. Musco, A. Schirone, G. Armiento, R. Danovaro, A. Dell'Anno

**Affiliations:** ^1^ Department of Life and Environmental Sciences Polytechnic University of Marche, UNIVPM Ancona Italy; ^2^ National Biodiversity Future Centre Palermo Italy; ^3^ Department of Research Infrastructures for Marine Biological Resources Stazione Zoologica Anton Dohrn, Fano Marine Centre Fano Italy; ^4^ Department of Materials, Environmental Sciences and Urban Planning UNIVPM Ancona Italy; ^5^ Department of Biological and Environmental Sciences and Technologies Salento University Lecce Italy; ^6^ Department for Sustainability ENEA Santa Teresa Research Centre Pozzuolo di Lerici Italy; ^7^ Department for Sustainability ENEA Casaccia Research Centre Roma Italy

**Keywords:** chronosequence, extracellular DNA, marine sediments, microbial genetic signatures, pollution, volcanic eruptions

## Abstract

Coastal marine ecosystems face escalating threats from multiple anthropogenic stressors, including the release of industrial contaminants. Despite decades of industrial activity impacting marine ecosystems, long‐term effects on microbial communities and related key ecological functions remain unclear. Here, we analysed the prokaryotic genetic signatures of extracellular DNA preserved in sediment layers dating from the mid‐17th century to the present day collected from two sites of one of the most polluted European coastal areas (i.e., Bagnoli‐Coroglio Bay, Tyrrhenian Sea), where industrial activities began in the early 20th century and ended in 1992. Archaeal 16S rDNA copy numbers were higher than bacterial ones, reaching values of 2.3 and 8.8 × 10^7^ copies during pre‐industrial volcanic episodes and the intense industrial development period, respectively. Most of the archaeal genetic signatures identified along the sediment vertical profiles belong to Bathyarchaeia. The pre‐industrial period showed lower diversity in terms of Amplicon Sequence Variants (ASVs) belonging to 70 prokaryotic families when compared with industrialisation periods (182 families), suggesting prokaryotic ability to respond and change in relation to modified environmental conditions occurring over time. High microbial β‐diversity values were observed, with major shifts occurring for more than 50 prokaryotic taxa in both cores, suggesting that chemical contamination and volcanic eruptions fostered microbial succession, selecting certain taxa more adapted to cope with such adverse ecological conditions. Our findings indicate that extracellular DNA pools of marine sediments can hold information on long‐term changes in benthic microbial diversity, representing valuable archives for understanding ecosystem dynamics over time.

## 1 Introduction

The Earth is rapidly changing due to increasing anthropogenic pressures (Steffen et al. 2015). Although there is evidence of ongoing change in the biosphere, it is still unclear how the Anthropocene has altered marine and terrestrial ecosystems beyond natural multi‐decadal to centennial variability (Kidwell 2015; Kuwae et al. 2024; Lewis and Maslin 2018). This is mainly due to the lack of long‐term observational time series, going back to the pre‐industrial era (Jonkers et al. 2019).

Over the 20th century, many coastal ecosystems have experienced increasing anthropogenic activities leading to unprecedented changes in biodiversity loss and ecosystem functions (Cardinale et al. 2012; He and Silliman 2019; Luypaert et al. 2020). It is known that changes in microbial communities can alter the stability and resilience of the ecosystem, affecting benthic organisms (Ranheim Sveen et al. 2024; Xu et al. 2025).

Marine sediments host abundant and diverse prokaryotic assemblages (Baker et al. 2021; Danovaro et al. 2015; Flemming and Wuertz 2019), which are key players in ecosystem functioning by controlling processes relevant for global biogeochemical cycles (Danovaro et al. 2016; Hoshino et al. 2020; Underwood et al. 2022; Zou et al. 2025). Despite the advances in our knowledge of prokaryotic diversity and the ecological functions they mediate (Grossart et al. 2019; Laiolo et al. 2024; Van Den Bossche et al. 2025), information on changes in benthic microbial assemblages occurring over time in relation to natural and anthropogenic pressures (e.g., pollution, climate change, habitat destruction) is still limited (Nguyen et al. 2023; Zhu and Penuelas 2020). Previous studies have shown contrasting results regarding the impact of anthropogenic activities due to pollutants on benthic prokaryotic assemblages, either by reporting no effects or a decrease in diversity (Jeanbille et al. 2016; Quero et al. 2015; Tamburini et al. 2020), through the selection of resistant taxa able to tolerate and/or metabolise contaminants (Nogales et al. 2011). At the same time, it remains unclear how prokaryotes inhabiting coastal sediments can respond to impacts due to natural episodic events such as storms, river runoffs and volcanic eruptions (Ares et al. 2020; Fazi et al. 2020; Pickarski et al. 2023; Rolfes et al. 2025).

Changes over time of benthic microbial assemblages have been assessed at different temporal scales by molecular analyses carried out on total DNA pools isolated from dated sediment cores and targeting diverse taxonomic groups (Barrenechea Angeles et al. 2023; Coolen et al. 2013; Hoshino and Inagaki 2024; Li et al. 2024; Nguyen et al. 2023; Vuillemin et al. 2019). The extracellular DNA pool, released into marine sediments following cellular death, comprises the majority of the total DNA pool, exceeding the amount of DNA contained in the living biomass by up to tenfold (Dell'Anno et al. 2002; Dell'Anno and Danovaro 2005). It can persist for thousands of years depending on a variety of physicochemical factors, which limit/avoid degradation by DNase activity (Nielsen et al. 2007; Pietramellara et al. 2008; Torti et al. 2015; Ye et al. 2022). As such, the analysis of the genetic signatures within extracellular DNA pools preserved in dated sediments holds great potential for identifying previously present taxa and for advancing our understanding of the prokaryotic succession in response to natural and human‐related stressors (Balint et al. 2018; Ellegaard et al. 2020; Fordham et al. 2020).

In the present study, we quantified and characterised prokaryotic genetic signatures preserved in extracellular DNA from sediment layers dated from the mid‐17th century to the present, in relation to the potential impacts due to past natural and anthropogenic events. To do so, we identified the benthic sites of Bagnoli‐Coroglio bay (Gulf of Naples, South Tyrrhenian Sea) as a model, since they were subjected to past disturbance events related to ash deposition from Vesuvian eruptions and to the discharge of huge amounts of chemical contaminants by industrial activities started at the beginning of the 20th century and ceased in the early 1990s (Morroni et al. 2020; Romano et al. 2004, 2018; Tangherlini et al. 2020; Trifuoggi et al. 2017).

Findings reported in the present study, based on the genetic imprints present in the extracellular DNA, provide new insights into potential resistant and sensitive microbial taxa to natural and anthropogenic disturbance events and can represent a benchmark for improving our knowledge of microbial succession occurring over time.

## 2 Materials and Methods

### 2.1 Study Areas and Sampling Strategy

The Bagnoli‐Coroglio area, located in the Gulf of Naples (Southern Tyrrhenian Sea), is classified as a Site of National Interest due to extensive chemical contamination from past industrial activities. Industrial operations, primarily a steel plant processing fossil coal, iron and limestone, began in 1905 and ceased in the early 1990s (De Vivo and Lima 2008; Qu et al. 2018). Previous studies have documented elevated concentrations of metals and polycyclic aromatic hydrocarbons (PAHs) in marine sediments adjacent to the former plant, directly linked to these industrial discharges (Albanese et al. 2010; Arienzo et al. 2017; De Vivo and Lima 2008; Romano et al. 2009, 2018; Trifuoggi et al. 2017).

Two sediment cores, hereafter named core 1 and core 2, respectively, were collected in the Bagnoli‐Coroglio bay on 5 December 2018 through a SW‐104 gravity corer, which guarantees the collection of undisturbed sediments (Figure S1).

Core 1 was collected in an adjacent area of a dismissed petrochemical plant, at a distance of ca. 1.7 km, whereas core 2 was located approximately 4 km from the former industrial site and characterised by lower anthropogenic inputs.

The sediment cores were dissected at the laboratories in sterile conditions and with a resolution of 1 cm up to depths of 60 cm for core 1 and 50 cm for core 2 and stored at −20°C until further processing. All sediment layers were analysed for chronological reconstruction (see below). On the basis of geochronology, we selected sediment layers from the two cores for chemical (contaminants) and molecular analysis (Figure S2).

**FIGURE 1:**
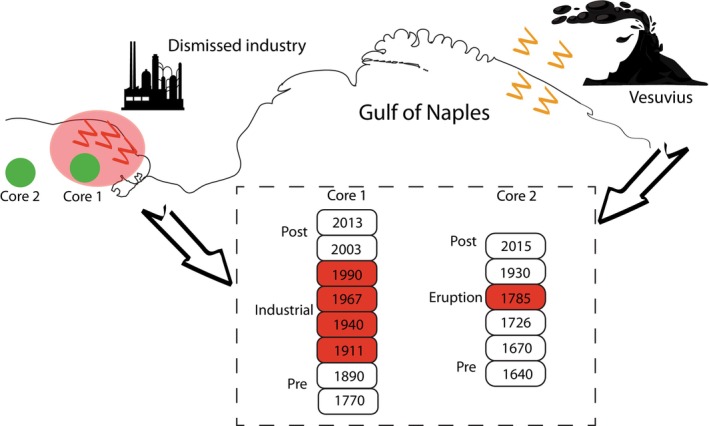
Overview of the selected layers (highlighted in the dashed rectangle) of two sediment cores collected from the Bagnoli‐Coroglio Bay. Core 1 was useful to assess the consequences on the microbial communities of the entire industrialisation period (1911–1992) ended with the decommissioning of the industrial site, whereas the analyses of core 2 were aimed at investigating the microbial responses to a period of intense activity of Vesuvius occurred between 1764 and 1794, including a major eruption in 1779.

The sediment layers selected from core 1 (i.e., 1–2, 5–6, 9–10, 13–14, 17–18, 23–24, 27–28, 53–54 cm) allowed us to compare the prokaryotic abundances and diversity in relation to different historical periods, corresponding to different contaminant inputs (pre‐industrial, industrial, post‐industrial; Figure 1). The sediment layers selected from core 2 (i.e., 1–2, 15–16, 29–30, 35–36, 41–42, 47–48 cm) were used to investigate the potential impact due to the deposition of volcanic material resulting in the sub‐cycles of Vesuvian eruptions that occurred between 1764 and 1794, characterised by eruption columns reaching up to 15 km in height (Scandone and Gasparini 1995). Indeed, based on the estimated mass accumulation rates (0.18 ± 0.01 g cm^−2^ for core 1 and 0.11 g cm^−2^ for core 2), core 2 captured a longer temporal record, allowing the investigation of older sediment layers and extending the reconstruction of past environmental changes further back in time. Finally, layers from the two cores characterised by similar age, considering the uncertainty of the radiometric dating method, were compared to assess microbial responses under different levels of contamination.

### 2.2 Geochronology of Sediment Layers

For sediment layers dating, gamma spectrometry analyses were carried out using 20 g of samples to evaluate the activities of ^210^Pb and ^226^Ra, according to procedures reported in (Delbono et al. 2016). The excess ^210^Pb (^210^Pb_ex_) activity was computed by finding the difference between the total ^210^Pb and the fraction in equilibrium with the parent radionuclide ^220^Rn; this value was estimated by the almost constant differences between ^210^Pb and ^226^Ra in the deepest part of the cores (Armiento et al. 2022). To account for sediment layer compaction, mass depth (g cm^‐2^) was used.

To set a date of each sediment core layer, a Constant Rate of Supply (CRS) model was applied (Appleby and Oldfield 1978) for ^210^Pb. For core 2, the dating of the layers from 1640 to 1930 was modelled by taking into account the post‐depositional processes recorded (Buffoni et al. 2020). The model was validated by the high levels of ^226^Ra useful for identifying the documented sub‐cycles of Vesuvius activities recorded in the 1764–1779 and 1783–1794 (Armiento et al. 2022; Scandone and Gasparini 1995; Voltaggio et al. 2004). The estimated age of the sediment layers of the two cores is reported in Table S1.

### 2.3 Determination of Metals and Polycyclic Aromatic Hydrocarbons

Analyses for determining metals (arsenic, cadmium, chromium, copper, mercury, nickel, lead, zinc) and PAHs concentrations were performed on sediment layers following the procedures previously described (Armiento et al. 2020; Barrenechea Angeles et al. 2023). These compounds are the main cause of site contamination (Armiento et al. 2020). To estimate the contamination levels in dated cores, the mERMq (Kowalska et al. 2018; Tangherlini et al. 2020) was calculated for both heavy metals (Table S1) and PAHs (Table S2) based on the available ERM values for these contaminants (Long et al. 2000, 1995). Values of mERMq were considered as follows: values ≤ 0.1 indicate no expected adverse biological effects; values between 0.1 and 0.5 suggest the potential for adverse effects; values from 0.5 to 1.5 reflect a moderate likelihood of adverse effects; and values > 1.5 indicate a high probability of significant biological impact (Kowalska et al. 2018; Long et al. 2000).

### 2.4 Extraction of Extracellular DNA Pool

Extracellular DNA isolated from the selected sediment layers (*n* = 2) was carried out as described by Corinaldesi et al. (2005). This isolation procedure has been shown to be robust and reliable, excluding any intact cells and potential contaminations from cell lysis of microbial cells (Corinaldesi et al. 2005). Briefly, 2.5 g (wet weight) of each sampled core slice was added to 7.5 mL of 0.1 M sodium phosphate buffer (pH 8.0) with 0.5 g of acid‐washed polyvinylpolypyrrolidone (0.05% final concentration). Samples were manually shaken for three 1‐min cycles, with 1 min of cooling on ice between cycles. Afterwards, sodium dodecyl sulphate (final concentration, 0.1%) was added, and the samples were shaken again for 10 s. Samples were then incubated on ice and centrifuged at 500 *g* for 10 min at 4°C, and the supernatants were transferred to new sterile tubes. The sediment pellets were washed twice by adding 7.5 mL of 0.1 M sodium phosphate buffer (pH 8.0) and centrifuged as described above. Supernatants of each sample were pooled together and centrifuged for 20 min at 10,000 *g* (at 4°C). After centrifugation, the supernatants containing extracellular DNA were filtered through 0.02‐μm syringe filters (Whatman, Anotop, Merck KGaA, Darmstadt, Germany) to remove any contaminating viruses or cells. The extracellular DNA was precipitated by adding 1 volume of a cetyltrimethylammonium bromide (CTAB) solution (1% CTAB in 50 mM Tris 10 mM EDTA, pH 8.0). Samples were incubated at 65°C for 30 min and then centrifuged at 5000 *g* for 10 min at 4°C. The supernatants were discarded, and each pellet was resuspended in high‐salt TE buffer (10 mM Tris–HCl, 0.1 mM EDTA, 1 M NaCl; pH 8.0). Then 0.6 volume of cold isopropanol was added to each sample, and the samples were incubated overnight at −20°C and centrifuged at 10,000 *g* for 15 min at 4°C. The pellets containing extracellular DNA were resuspended in 150 μL of sterile ultrapure water filtered through 0.02‐μm‐pore‐size membrane filters and purified through DNeasy PowerClean Cleanup Kit (Qiagen, Hilden, Germany) according to manufacturer's instructions. Extracellular DNA concentration was estimated by measuring absorbance at 260 nm and the purity by 260/280 and 260/230 nm ratios, through a NanoDrop spectrophotometer (ND‐1000 UV–Vis Spectrophotometer; NanoDrop Technologies, Wilmington, DE, USA). The fragment lengths of extracellular DNA were evaluated with TapeStation 4200 system (Agilent, Santa Clara, CA) with D1000 ScreenTape assay (Figure S3A–D).

### 2.5 Bacterial and Archaeal 16S rDNA Gene Copies Number Quantification

Real‐time PCR (qPCR) analyses were carried out to quantify the prokaryotic gene copies number within the extracellular DNA pools isolated from surface to subsurface dated sediment layers. SsoAdvanced Universal Probes Supermix (BioRad, Hercules, CA, USA) was used for the quantification of gene copy numbers on CFX Connect Real‐Time PCR detection system (BioRad, Hercules, CA, USA). The amplification reactions were performed in a final volume of 10 μL in film‐sealed optical 96‐well qPCR plates. Each reaction contained the SsoAdvanced Universal Probes Supermix, 0.5/0.2 μM (for bacterial) or 0.8/0.2 μM (for archaeal) primers/probes, respectively, and 1 μL of template DNA.

The prokaryotic 16S rDNA sequences contained within the extracellular DNA pools were amplified using primers and probes selectively targeting Archaea (Arch349F: 5′‐GYGCASCAGKCGMGAAW‐3′, Arch806R: 5′‐GGACTACVSGGGTATCTAAT‐3′ and probe Arch516F: 5′‐TGYCAGCCGCCGCGGTAAHACCVGC‐3′) (Takai and Horikoshi 2000) and Bacteria (Bac331F: 5′‐TCCTACGGGAGGCAGCAGT‐3′, Bac772R: 5′‐GGACTACCAGGGTATCTAATCCTGTT‐3′ and probe 5′‐CGTATTACCGCGGCTGCTGGCAC‐3′, targeting *Escherichia coli* 16S rDNA region from position 506 to 528) (Nadkarni et al. 2002). All probes used for qPCR TaqMan assay were assembled with a fluorescent reporter dye (6‐FAM) in the 5′ position and a Black Hole Quencher 1 (BHQ‐1) in the 3′ position, which ensures the specificity of the fluorescent signal generated only from the amplification of the target sequence (Smith and Osborn 2009).

The adopted thermal cycling conditions were: 3 min at 95°C, followed by 40 cycles of 15 s at 95°C and 1 min at 60°C for Bacteria; 3 min at 95°C, followed by 40 cycles of 15 s at 95°C and 5 min at 57°C for Archaea. The presence of a single PCR product of the expected size was checked using 1% agarose gel electrophoresis. The CFX Manager software version 3.1 was used to calculate Cq, efficiency (*E*) and *R* ^2^ values of standard curves for each plate and to determine the sample concentrations based on the standard curves. Calibration curves were included in all reactions, with six dilutions of samples with known concentrations of gene copy numbers obtained from *E. coli* (for Bacteria) and *Methanocaldococcus jannaschii* (for Archaea), according to the MIQE guidelines (Bustin et al. 2009). The values were plotted against the number of cycles at which the fluorescence signal increased above background, or the cycle threshold (the Ct value) of samples. To exclude potential qPCR bias due to the presence of inhibitors, reactions were run using undiluted aliquots of DNA isolated from the sediment layers, in addition to running all sample extracts in serial 10‐fold dilutions. The log‐linear relationship between Ct and the dilution factor was observed in all of the samples by using the 10‐fold dilution (Cao et al. 2012; Lloyd et al. 2010). The copy number of gene sequences determined by qPCR was normalised to sediment dry weight (gr) and DNA extracted (ng). All samples, standards and negative controls were run in triplicate. Analyses of variance (ANOVA) were carried out to test for differences in bacterial and archaeal 16S rDNA copy numbers among layers of both cores. Where significant differences occurred, Tukey's honest significant difference test was carried out to identify differences among layers. Prokaryotic copy numbers quantified in specific dated layers of two cores were compared using *t*‐tests after checking the normality of data with the Shapiro–Wilk test.

### 2.6 Sequencing and Bioinformatics

Three aliquots of extracellular DNA of each sample were used for library preparation using the primer pairs 515F–Y (5′‐GTGYCAGCMGCCGCGGTAA) and 926R (5′CCGYCAATTYMTTTRAGTTT) (Parada et al. 2016), followed by sequencing on a single Illumina MiSeq flowcell V3 2 × 300bp (Illumina, San Diego, CA) by LGC Genomics GmbH (Berlin, Germany). Paired‐end sequences were demultiplexed within QIIME2 (Bolyen et al. 2019). Primers from paired‐end sequences were removed through the cutadapt plugin with an error rate of 0.1 (M. Martin 2011). Reads were truncated at position 261 for the forward reads and at position 177 for the reverse reads to exclude low‐quality bases (*Q* < 30). Reads were subsequently trimmed and denoised using default parameters of DADA2 (Callahan et al. 2016) to produce a table with the sequence abundance of all the Amplicon Sequence Variants (ASVs). A subset of the SILVA database (release 138; Quast et al. 2013) was created through the extract‐reads procedure within QIIME2 by trimming the whole database to the region amplified by the primers used; sequences from the subsetted database were then used as an input for subsequent classification steps carried out on the representative sequences using the classify‐consensus‐vsearch tool within QIIME2, with default parameters (Rognes et al. 2016). The resulting abundance table and the taxonomic information were both loaded within R (version 4.3.2) through the RStudio interface (Allaire 2012) and analysed using the Microeco (Liu et al. 2021) package. Taxonomic information was used to remove eukaryotic, chloroplast and mitochondrial‐related sequences from the ASV list and the ASV abundance table before subsequent analyses. To exclude the influence of variability in sequencing effort affecting both α and β‐diversity metrics, the filtered abundance table was subsequently rarefied to a minimum common number of sequences (10,100) and then split between cores (Schloss 2024). The rarefied tables were used to compute α‐diversity (i.e., ASV richness, Shannon, Pielou, Simpson) and β‐diversity (based on Bray‐Curtis distances between samples) through the Microeco package. Venn diagrams for each core were generated to calculate the number of ASVs shared between different periods (referring either to the industrialisation of the area for core 1 and to the Vesuvius' eruption for core 2) and between layers identified with similar ages for evaluating the different levels of anthropogenic impact, together with their taxonomic information and sequence abundance. To infer the effect of the anthropogenic impacts or natural events on the ASV richness, we defined ‘resistant ASVs’ as those ASVs present across the entire core, ‘resilient ASVs’ as those detected before and after impact, and ‘sensitive ASVs’ as those present before and during the industrialisation period of the Bagnoli‐Coroglio bay or the Vesuvius eruption; the relative abundance of reads affiliating with these ASVs was calculated after aggregating ASVs of the same defined group. The statistical significance of the clustering of samples after ordination for β‐diversity analysis was assessed through a PERMANOVA test within Microeco.

To evaluate the impact of different variables (such as sediment age and pollution levels) on the prokaryotic assemblage structure, we applied different statistical approaches. First, to identify significant differences in the relative abundances of ASVs classified at family level, we compared their relative abundances across the different periods identified in both cores, as well as between layers of two cores with similar ages, using the marginal effects at the mean (MEM) approach through the emmeans_test and Dunn's Kruskal–Wallis multiple comparisons within the package emmeans in R (Lenth 2025). We also carried out Mantel test correlations between variables and the distance matrices obtained during β‐diversity analyses to gain insights into the potential effect of such parameters on inter‐sample differences; then, we analysed the relationships between inter‐sample Bray‐Curtis distances on the ASV tables and inter‐sample Euclidean distances on single parameters and evaluated significant relationships through regression analyses (Qiu et al. 2024). All plots were generated with ggplot2 in R (Wickham 2011).

## 3 Results

### 3.1 Chemical Contamination in the Sediment Cores

**FIGURE 2:**
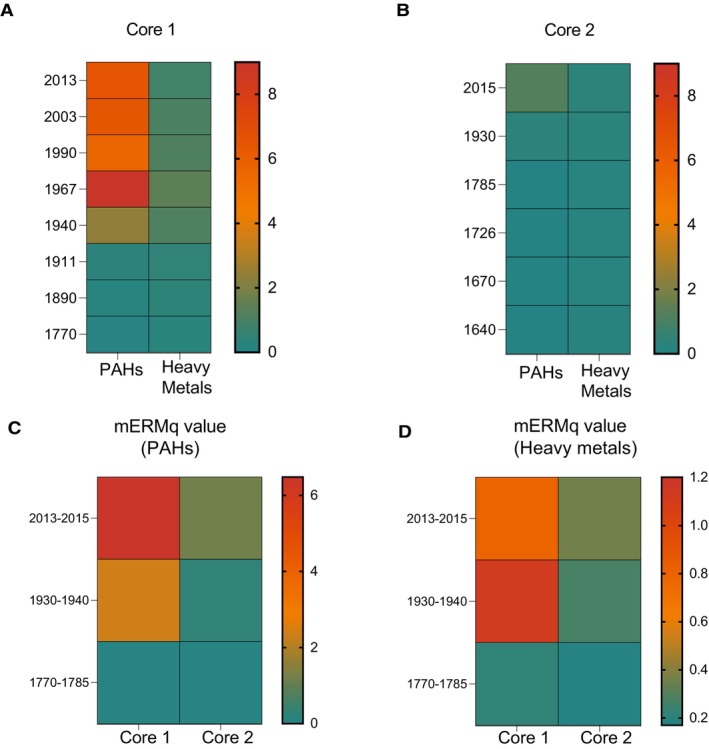
Heatmap showing a comparison of the mERMq values calculated for sediment layers in core 1 and core 2. Panels A and B display the mERMq values calculated for each selected layer based on the concentrations of polycyclic aromatic hydrocarbons (PAHs) and heavy metals in core 1 and core 2, respectively. The mERMq values calculated using PAHs of sediment layers with similar ages were reported in C, whereas mERMq based on heavy metals in D. Tiles are coloured according to the mERMq value for the pollutant class.

The core 1 exhibited higher mERMq values of PAHs and heavy metals than core 2 (Figure 2A,B).

The mERMq values calculated based on PAH concentrations ranged from 0.02 to 8.99 in core 1 and from 0.01 to 1.18 in core 2. The highest values of mERMq for PAHs were detected in the sediment layer of core 1, dated to 1967, which corresponds to the peak of industrial expansion in the area (Table S2). The mERMq values calculated on the basis of metal concentrations displayed values ranging from 0.21 to 1.39 within core 1 and from 0.15 to 0.35 within core 2 (Table S1). Sediment layers corresponding to both the post‐industrial (2013–2015) and the industrial period (1930–1940) were characterised by mERMq values much higher than those of the pre‐industrial period (Figure 2C,D).

### 3.2 Bacterial and Archaeal 16S rDNA Copy Numbers

**FIGURE 3:**
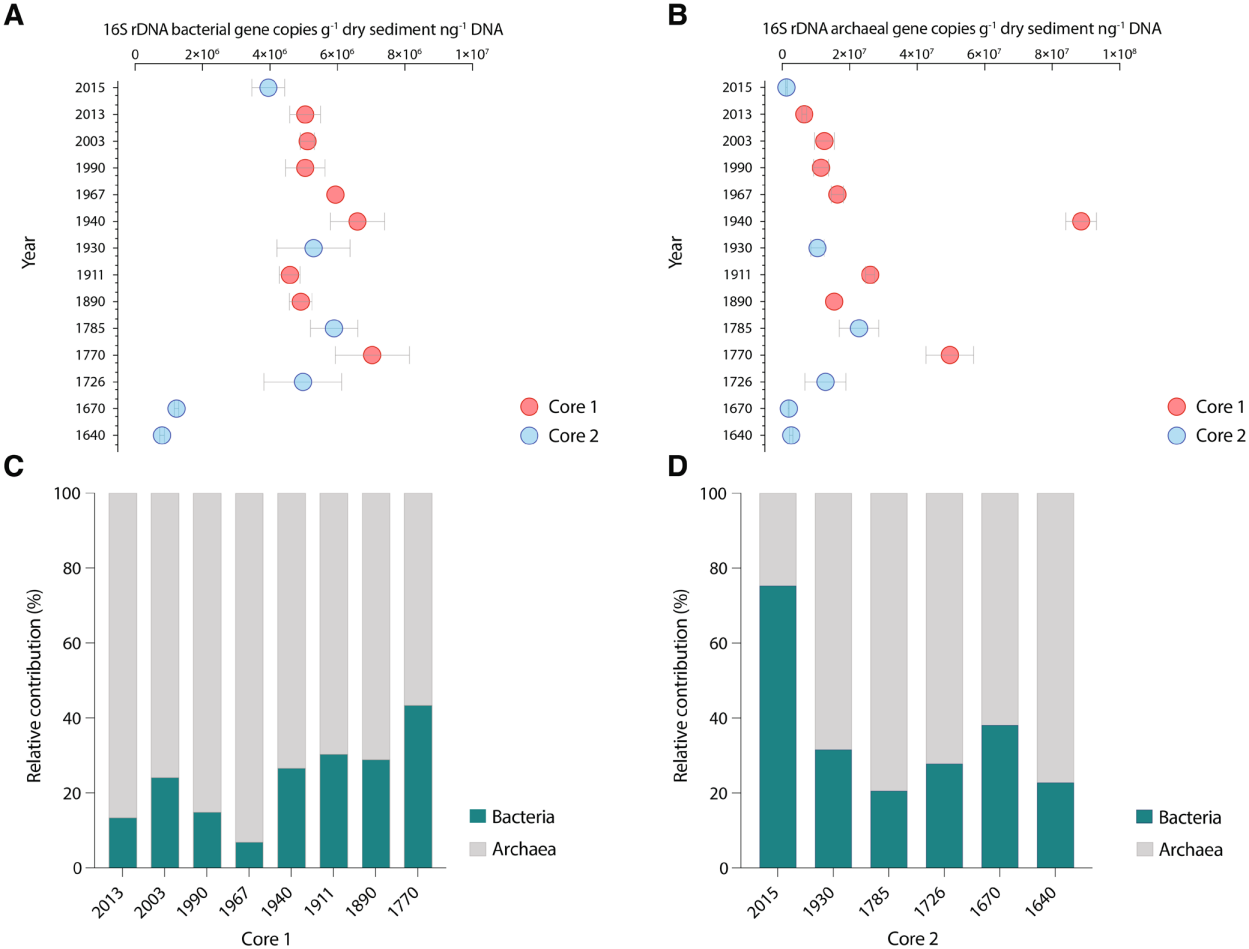
Abundances of extracellular bacterial (A) and archaeal (B) 16S rDNA copy numbers expressed as copies g^−1^ sediment DNA extracted and quantified from core 1, showing high levels of contaminants and core 2 characterised by low concentrations of PAHs and heavy metals. Error bars indicate ± SE. Relative contributions of bacterial and archaeal 16S rDNA copy numbers in each layer analysed of core 1 (C) and core 2 (D).

Bacterial and archaeal 16S rDNA copy numbers contained in the extracellular DNA pools were quantified by qPCR in the dated layers of both cores analysed (Figure 3). Bacterial and archaeal 16S rDNA copy numbers displayed a hump‐shaped distribution along the sediment profiles. Bacterial 16S rDNA copy numbers (range: 8.0 ± 0.7 × 10^5^–7.0 ± 1.1 × 10^6^; Figure 3A) were always lower than those of archaea (range: 1.3 ± 0.2 × 10^6^–8.8 ± 0.4 × 10^7^ copies; Figure 3B), except for the surficial sediment layer (0–2 cm) of core 2. As such, the contribution of the extracellular archaeal 16S rDNA copies accounted for up to 93% of the total pool of prokaryotic sequences in core 1 in 1967 (as the sum of the number of the archaeal and bacterial 16S rDNA copies contained in the extracellular pool; Figure 3C) and up to 77% in core 2 (Figure 3D).

The highest number of archaeal 16S rDNA copies was observed in core 1, during the developing industrial phase (1940), with significant differences when compared to the other periods (i.e., sediment layers analysed within the same core; ANOVA *F*_7,40_ = 62.72, *p*‐value < 0.001; Tables S3 and S4). Bacterial copy numbers showed significant differences among sediment layers in core 2, but such differences were weaker in core 1 (ANOVA *F*_7,40_ = 2.31, *p*‐value > 0.05; Tables S3 and S5). In 1785, during an intensive recorded volcanic activity, archaeal and bacterial gene abundances reached values of 2.3 ± 0.6 × 10^7^ and 5.9 ± 0.7 × 10^6^, respectively, in core 2 and significantly higher than those recorded in older layers analysed (1640 and 1670) (*p*‐value < 0.05). The number of archaeal 16S rDNA copies was significantly higher in the 1940 and 2013 layers of core 1 compared to the corresponding layers (1930 and 2015, respectively) in core 2 (*t*‐test; *p*‐value < 0.001; Table S6).

### 3.3 Prokaryotic Diversity Within the Extracellular DNA Pools

MiSeq runs carried out on prokaryotic genes isolated from dated cores resulted in a total of 4.23 million raw paired reads, reduced to 2.05 million after removal of the low‐quality sequences (Table S7). Rarefaction curves indicate that the sequencing depth was sufficient to capture the ASV diversity across all analysed sediment layers (Figure S4).

A total of 17,506 ASVs were identified throughout core 1, whereas 2170 ASVs were detected across core 2. Analysis of the relative abundance of ASVs at Class level identified a total of 178 classes or higher taxonomic ranks, mostly belonging to Desulfobacteria (10%), Gammaproteobacteria (6.6%), Bathyarchaeia (5.4%) and Dehalococcoidia (3.8%) (Figure S5). In particular, the average abundances of Gammaproteobacteria ranged from 2.4% to 23.1% in core 1 and from 9.9% to 23.9% in core 2; Dehalococcoidia ranged from 1.4% to 17.2% in core 1 and from 0.8% to 19.7% in core 2; Bathyarchaeia ranged from 2.4% to 14.7% in core 1 and from 3.2% to 15.4% in core 2; and Desulfobacteria ranged from 6.0% to 11.4% in core 1 and from 7.5% to 16.3% in core 2.

**FIGURE 4:**
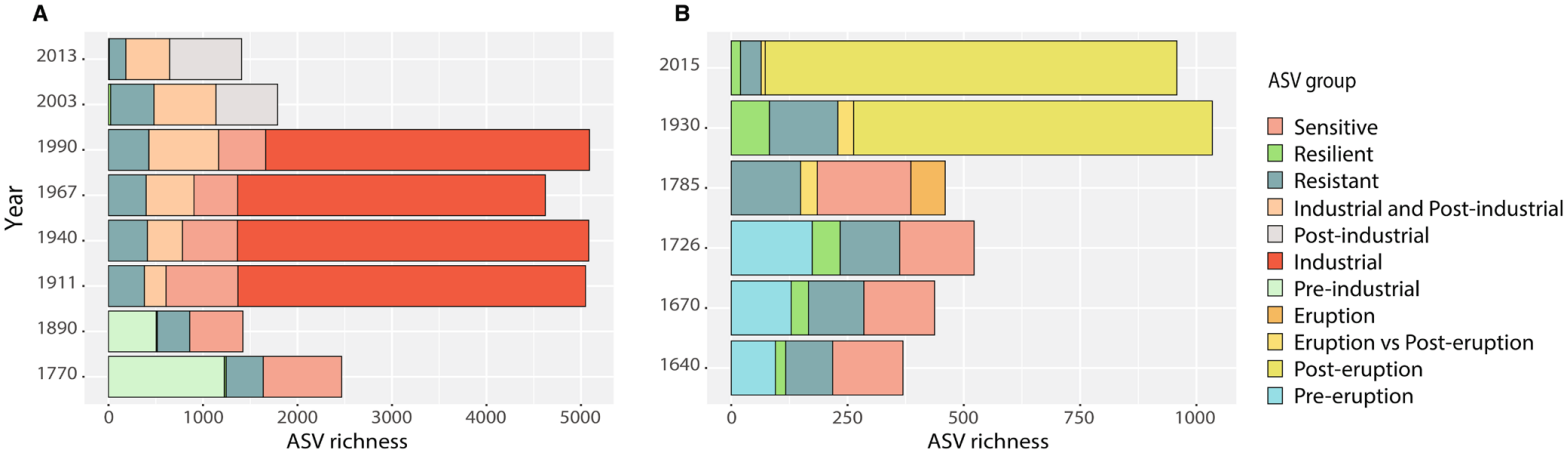
Histograms showing comparisons of prokaryotic ASV richness detected in layers with similar ages collected from two cores (A); Cumulative taxa barplots represent the number of ASVs present across the entire core (‘resistant’), detected before and after industrialisation (‘resilient’), present before and during the industrial period (‘sensitive’), ASVs shared between post‐industrial and industrial period and ASVs exclusively found during the periods identified (B).

In core 1, the highest number of ASVs was observed during the industrial period (range: 4622 to 5090 ASVs; Figure 4A). In core 2, the highest ASV richness was recorded in the layer dated to 1930 (Figure 4B), although with a lower value (1034 ASVs) than core 1, and twice as high as in the older layers. Similar patterns were observed for the Shannon diversity index, which displayed in core 1 the highest values in the industrial period (on average 7.4 ± 0.4), and in core 2 in the sediment layer dated to 1930 (Table S8).

**FIGURE 5:**
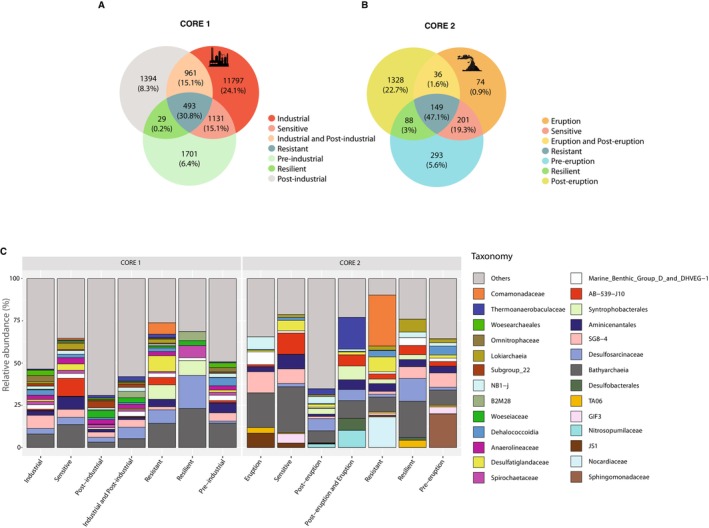
Analyses of ASVs shared between different periods for identifying resistant (light blue), sensitive (pink) and resilient (green) prokaryotic genetic signatures. Venn diagrams represent the ASV abundance and sequence contribution (as percentage of reads identified in each core) of samples in different phases identified in the core 1 (pre‐industrial, industrial, post‐industrial); (A) and 2 (pre‐eruption, eruption and post‐eruption); (B) highlighted with different colours. Taxa bar plots representing a taxonomic affiliation at Family (whenever possible) or Order/Class level of relative abundance of ASVs identified in different periods within both cores (C).

In core 1, 1701 ASVs were exclusively found in the sediment layers of the pre‐industrial period (6.4% of the total sequences resulted from core 1); 11,797 ASVs were exclusive to layers corresponding to the industrial period (24.1%); and 1394 ASVs were only detected in layers after the end of the industrial activity (8.3%; Figure 5A). In core 2, 293 ASVs were detected exclusively in sediment layers dated before the intensification of Vesuvius eruptions (5.6% of the sequences resulted from core 2); 74 ASVs were exclusive to eruptive periods (0.9%); and 1328 ASVs were only found in periods after eruptive events (22.7%; Figure 5B).

In core 1, 493 ASVs were always present (here named ‘resistant ASVs’) in the different periods we considered (pre‐industrial, industrial and post‐industrial) and accounted altogether for a relevant fraction of the total sequences of such core (ca. 31%). In core 2, 149 ASVs were shared among pre‐eruptive, eruptive and post‐eruptive periods (i.e., ‘resistant ASVs’), accounting for ca. 47% of the total sequences. In core 1, the ‘resistant ASVs’ (ranging from 175 in 2013 to 458 in 2003; Table S9) mainly belonged to Bathyarchaeia (14.3% of reads grouped as resistant; Figure 5C), whereas in core 2 (ranging from 44 in 2015 to 149 in 1785; Table S10) the ‘resistant ASVs’ were mainly assigned to the family *Comamonadaceae* (30.2% of resistant). In core 2, ASVs belonging to Bathyarchaeia were detected across all historical phases, with a relative abundance of 8.6%. ‘Sensitive’ ASVs affiliated with Aminicentales were detected before and during the industrial period, accounting for 7.5% of the reads resulting from that period, along with *Desulfatiglandaceae* (3.8%) and *Anaerolineaceae* (3.7%). The ASVs shared between pre‐industrial/volcanic and post‐industrial/volcanic periods (‘resilient’) were 29 in core 1 and 88 in core 2, accounting for 0.2% and 3% of the total reads identified in each core, respectively. Bathyarchaeia and *Desulfosarcinaceae* were the most abundant resilient taxa in both cores, together accounting for 42.7% of the total 'resilient sequences' identified in each core.

**FIGURE 6:**
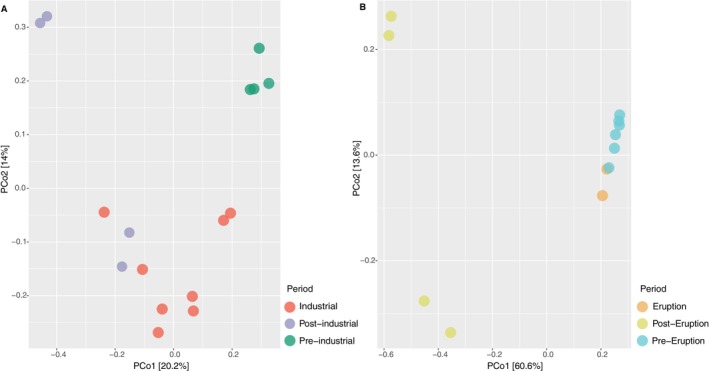
PCoA plots of Bray–Curtis dissimilarity showing the ordination of samples belonging to dated layers of core 1 (A) and core 2 (B). Each sample is depicted as a point according to the period: pre‐industrial (green circles), industrial (red circles) and post‐industrial (violet circles). Pre‐eruption (light blue circles), eruption (orange circles) and post‐eruption (lime circles).

**FIGURE 7:**
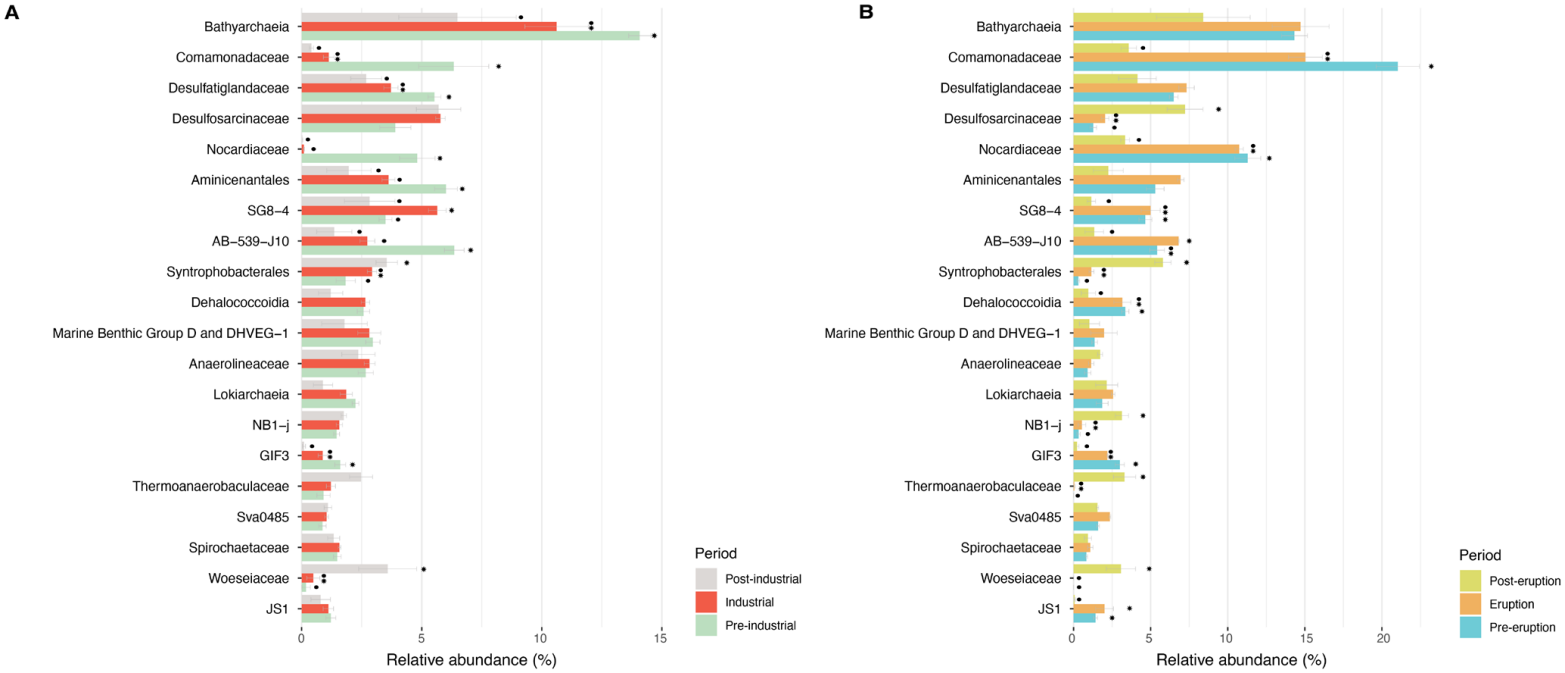
Bar plots showing the relative abundance (%) of the 20 most represented prokaryotic taxa across different historical periods identified in core 1 (A) and 2 (B), reported in different colours. Bars represent the mean relative abundance of sequences classified at family or higher taxonomic levels across samples of the corresponding period. Error bars indicate ± standard deviations. Different symbols (asterisks and circles) indicate statistically significant differences between periods (*p* < 0.05).

**FIGURE 8:**
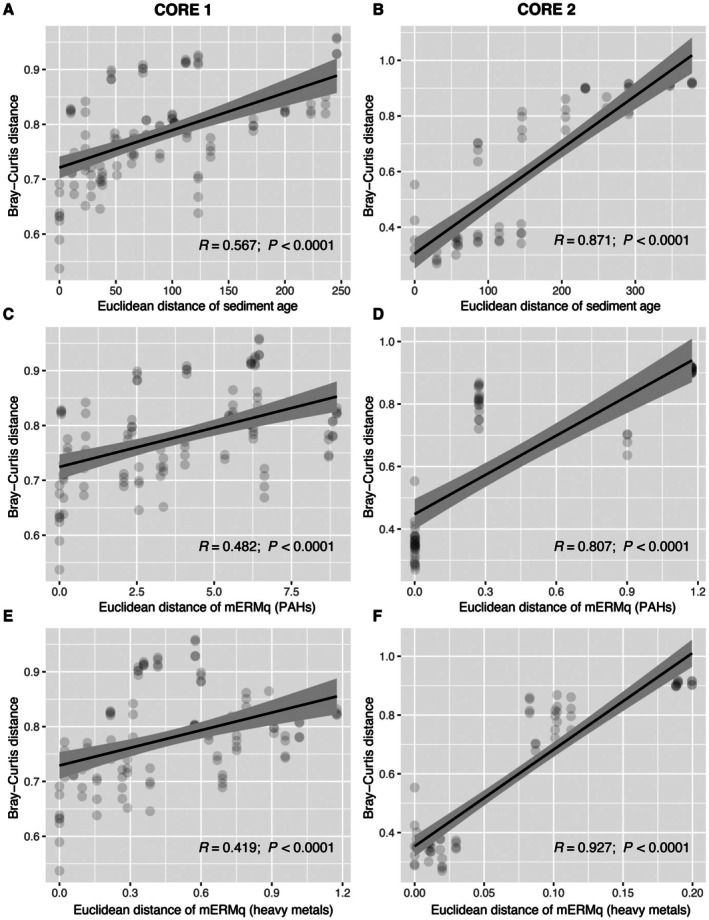
Scatterfit plots showing the relationships between Bray‐Curtis distances of prokaryotic assemblage composition (in terms of relative abundance values of ASVs) and the Euclidean distances of age (A, B), mERMq of PAHs (C, D) and mERMq of metals (E, F) determined between the different sediment layers investigated across the vertical profile. On the left side panel, data collected from core 1 are shown, whereas on the right side, the values of core 2 are displayed.

Statistical analysis highlighted differences in the composition of the genetic imprints of the extracellular DNA preserved in the different sediment layers corresponding to the different periods considered in both cores (*p*‐value < 0.05; Figure 6; Table S11). Several prokaryotic taxa changed significantly over time (57 taxa in the core 1 and 53 in the core 2; Figure 7; Table S12); in particular, the quantitative relevance of reads belonging to Aminicenantales and *Comamonadaceae* decreased from the industrial to the post‐industrial period, whereas SG8‐4 and *Woeseiaceae* increased considering the same time interval. *Woeseiaceae* also increased during the post‐volcanic period, whereas *Nocardiaceae* decreased. Significant and positive (Mantel tests, *p*‐value < 0.05; Table S13) correlations were found between inter‐sample distances based on Bray‐Curtis and Euclidean distances between ages of layers, mERMq values for PAHs and mERMq values for heavy metals. Scatter fit plots computed on this data showed that such correlations were > 0.8 in core 2 and > 0.4 in core 1 (Figure 8).

The ASVs shared between sediment layers of the two cores of similar age ranged from 329 to 490, accounting for ca. 46%–61% of the total reads encountered in samples belonging to a similar period in both cores (Figure S6). The relative abundance of ASVs belonging to the same family between sediment layers of similar age changed significantly. For example, the relative abundances of ASVs belonging to the family *Nitrosopumilaceae* were significantly lower (*p* < 0.001) in core 1 compared to core 2 during the industrial period, whereas an opposite significant pattern (*p* < 0.001) was observed in the post‐industrial period (Table S14).

## 4 Discussion

Here, we assessed for the first time the long‐term temporal changes of the benthic prokaryotic assemblages as a potential consequence of natural and anthropogenically mediated impacts, based on the analysis of the genetic signatures preserved within the extracellular DNA. In this perspective, the analysis of dated sediment cores from the Bagnoli‐Coroglio bay offered us a unique opportunity to investigate microbial responses in relation to past disturbance events related to pollutant discharges and ash deposition due to volcanic eruptions of Vesuvius. The analysis of the sediment profiles carried out in the present study revealed clear signatures of past pollutant discharges, especially in core 1, characterised by very high concentrations of PAHs and metals and related hazardous quotients (mERMq), closer to the major source of contamination (i.e., industrial plants now dismissed), as previously documented (Barrenechea Angeles et al. 2023; De Vivo and Lima 2008; Passaro et al. 2020; Romano et al. 2009, 2018). At the same time, the estimated age of the sediment layer (i.e., 29–30 cm below the sediment surface) of core 2 matches the period of sub‐cycles of Vesuvian eruptions and the ash deposition occurring in the bay (Scandone and Gasparini 1995).

Extracellular DNA pools released by dead organisms can persist for thousands of years in marine sediments (Coolen and Overmann 2007; Corinaldesi et al. 2008; Torti et al. 2015; Ye et al. 2022), but the complex biogeochemical transformations occurring over time can alter our ability to quantify and characterise the genetic signatures present therein. The degradation of extracellular DNA pools, for instance, can affect PCR amplification steps. Therefore, assessing fragment lengths of extracellular DNA isolated from environmental samples is an important step for determining the suitability of primer pairs and sequencing platforms. In our study, we found that the majority of extracellular DNA fragments were longer than 400 bp, and that such fragment lengths were consistently detected across all samples, regardless of the age of the sediments analysed (up to ~400 years). This result indicates that Bagnoli‐Coroglio sediments may favour the preservation of sufficiently intact extracellular DNA over some centuries, allowing us to exclude biases related to the use of primer pairs targeting 16S rDNA fragments (~400–500 bp) in qPCR and metabarcoding analyses. In the case of older sediment layers extending over several thousand years, the extracellular DNA pool could be composed of shorter fragments, limiting the effectiveness of amplicon‐based approaches (Holman et al. 2025). In such cases, shotgun metagenomics can be applied to reconstruct past biodiversity without the need for PCR amplification (e.g., in dated sediments from thousands to millions of years old; Capo et al. 2022). However, metagenomics applied to extracellular DNA could have a lower taxonomic resolution power for properly assessing prokaryotic taxonomic diversity compared to metabarcoding targeting specifically 16S rDNA fragments (Holman et al. 2025; Tessler et al. 2017), due to the presence of a high fraction of nucleic acids of eukaryotic origin (Corinaldesi et al. 2011).

This study expands previous knowledge on the quantitative relevance of archaeal and bacterial genes contained within preserved extracellular DNA pools of dated sediments (Corinaldesi et al. 2011, 2018; Nguyen et al. 2023; Siano et al. 2021). Interestingly, in the Bagnoli‐Coroglio sediments, archaeal 16S rDNA gene copy numbers dominated the extracellular DNA pools (on average, ca. 74%), exceeding bacterial genetic signatures along the vertical profile except for the surface sediment layer of core 2. Similar patterns have been previously reported from the analysis of extracellular DNA pools isolated from sediment layers down to 90 cm (Torti et al. 2018). Extracellular archaeal 16S rDNA copy numbers showed a marked increase in the sediment layer dated to 1930, corresponding to the period of intensive industrial activities, which has not been observed for extracellular bacterial 16S rDNA copies. This finding suggests that Archaea may be more sensitive to anthropogenic disturbance events than Bacteria (Dong et al. 2019), which, by impacting the microbial living components, can enhance the release of their DNA into the surrounding environment. High copy numbers of prokaryotic 16S rDNA within the extracellular DNA pools were also found in the sediment layer dated 1785, when natural disturbance events related to sub‐cycle eruptions of Vesuvius occurred. Such events may cause significant changes in marine ecosystems (S. C. Martin 2020; Zhang et al. 2017) by enhancing sedimentation rates of organic and inorganic matter via ash ballasting, which can greatly affect microbial communities (Longman et al. 2019; Rolfes et al. 2025).

While different studies have focused on isolating bacteria capable of degrading PAHs, very few have examined the impacts of contaminants on microbial communities in natural environments (Haritash and Kaushik 2009; Tamburini et al. 2020; Tangherlini et al. 2020), and even less is known on the effects/changes occurring over time. Although metabarcoding of extracellular DNA has been rarely used to date, this study revealed that it holds great potential to expand our knowledge of historical events that have led to prokaryotic changes over long timescales. This is due to bacterial death that contributes to the accumulation of necromass‐derived extracellular DNA pools in marine sediments (Torti et al. 2015).

Indeed, extracellular prokaryotic genetic signatures preserved in the sediment we analysed were not only high but also diversified. Our findings based on the analysis of extracellular DNA revealed multi‐decadal variations in prokaryotic richness (in terms of ASV abundance and Shannon index) at one of the most polluted marine European sites (Barrenechea Angeles et al. 2023), which has faced huge contaminant discharge over the last century, thereby chronically exposing marine microbial communities to pollutants. During the eight decades of the industrial period (1911–1990), a fivefold increase in ASV abundances was recorded in the highly contaminated sediments of core1. Such a highly diverse imprint of the prokaryotic extracellular genes suggests that chronic exposure to contaminants can affect prokaryotes belonging to several different taxa, thus profoundly modifying the resident prokaryotic assemblages, also by favouring specialised microbes capable of metabolising specific contaminants (Albarano et al. 2020; Dell' Anno et al. 2021).

Analyses of shared ASVs along the vertical profile of both cores revealed the presence of a relevant fraction of ‘resistant ASVs’ (from 31% to 47% of the total sequences of each core), most of them belonging to Bathyarchaeia that can thrive both at high and low contamination levels. These results corroborate previous findings on the metabolic capacity of Bathyarchaeia to fulfil their carbon and energy requirements under a variety of environmental conditions (Evans et al. 2015; Hou et al. 2023; Yu et al. 2018). Our results also indicate that only a few ASVs were exclusive to the eruption period, whereas a larger fraction of ASVs was ‘resistant taxa’, suggesting the presence of a ‘core’ community over time able to cope with major changes in environmental conditions even related to events of high ash inputs due to volcanic activity. We found that among the resistant taxa identified, *Comamonadaceae* was one of the most abundant families. Members of such a family can still be present and metabolically active in volcanic sediments as they are able to carry out anaerobic ammonium oxidation coupled with iron (Fe^3+^) reduction (Bao and Li 2017; Jung et al. 2019).

In this study, we identified prokaryotic genetic signatures only between 1770 and 1990 that we defined as ‘sensitive ASVs’, which did not show a prompt adaptation to changing conditions, as observed with members of Aminicenantales. The same results were observed for the families of *Anaerolineaceae* and *Desulfatiglandaceae* (identified in core 1 in the period 1770–1990), which can degrade hydrocarbons and can be sensitive to different levels of contaminants present over time (Dong et al. 2022; Han et al. 2021; Kniemeyer et al. 2007; Zehnle et al. 2023).

Very few ‘resilient ASVs’ have been identified in our dataset, indicating that the community changed over time, and the consequences after a large disturbance caused mainly by industrialisation on microbial diversity are still present. For instance, distinct environmental conditions may favour resilient taxa identified in both cores, such as sulphate‐reducing bacteria belonging to *Desulfosarcinaceae*, including members of hydrocarbon degraders, which can benefit from massive hydrocarbon inputs (Kleindienst et al. 2014). This can be supported by the high quantities of contaminants still detected in surficial sediments of both cores that rely on a slight natural recovery of the site, which needs a longer time and/or specific restoration plans. On the other hand, past natural and industrial events can determine an irreversible change in the microbial community at the Bagnoli‐Coroglio site.

Our results showed that the composition of extracellular prokaryotic genes in core 1 sediments varied widely across the different historical periods identified, indicating a high β‐diversity as a consequence of changing environmental conditions superimposed by pollution discharges. In contrast, core 2 exhibited similar prokaryotic genetic signatures between eruption and pre‐eruption periods, likely reflecting the persistence of similar taxa sensitive to the ash inputs from Vesuvius eruptions that occurred between the 17th and 18th centuries (Scandone and Gasparini 1995).

The high number of diversified genetic signatures contained in the extracellular DNA pool of core 1, detected only during the industrialisation period, suggests the presence of many taxa that could not survive to high levels of contaminants. The microbial shifts over time could be mainly driven by the presence of specific genetic signatures in the extracellular DNA pools belonging to polychlorinated biphenyl degraders, such as *Comamonadaceae* and *Woeseiaceae*, which may have failed to adapt to the high levels of contaminants (Liang et al. 2014; Matturro et al. 2023; Tillmann et al. 2005). In core 2, decreasing relative abundances of ASVs belonging to the *Nocardiaceae* from the eruption to the post‐eruption period suggest that its members can be the first colonisers of volcanic deposits, contributing to the ecological succession (Roy et al. 2025).

Some taxa were always detected with different relative abundances, suggesting their plasticity and ability to adapt over time (Cravo‐Laureau and Duran 2014). The historical variations of heavy metal and PAH inputs can reflect the differences in community structure over time, with high and low mERMq values observed in our study, shedding light on how the huge discharge that occurred in the 20th century influenced past microbial communities. It should be recognised that, while microbial community structure could be influenced by contaminants discharged during the industrial period, other environmental factors (e.g., temperature, nutrient availability) might also synergistically exert an influence on microbial community structure (Jeanbille et al. 2016; Lu et al. 2019; Ramirez et al. 2020).

Different levels of contamination observed in sediment layers of similar ages of the two cores can alter the microbial community by affecting key taxa involved in the nitrogen cycle in marine sediments, such as *Nitrosopumilaceae* (Wright and Lehtovirta‐Morley 2023), which exhibited higher replication rates in pristine sites compared to those with elevated pollutant levels (Chen et al. 2019).

On one hand, studies based on advanced molecular analyses have provided evidence for historical environmental changes driving shifts in eukaryotic community composition and hence information on the relative importance of specific environmental drivers over long timescales (Balint et al. 2018). On the other hand, this study reports novel evidence on the prokaryotic assemblages reconstructing the consequences of the huge amounts of industrial pollutants that have occurred over the past century in the Bagnoli‐Coroglio site, which has become one of the most polluted marine European sites. Consequently, this study reports the impact of multiple pollutants on the microbial communities of the coastal environments over a long‐time scale, thus improving our understanding and prediction of biodiversity loss and/or recovery. This work highlights the importance of identifying changes in the microbial assemblage in sediments and the relevance of specific taxa in extracellular DNA since they can have a domino effect on other biological ecosystem components, affecting the entire marine food web.

In this study, we provide evidence for the application of innovative molecular approaches in the development of environmental assessments useful for recovering degraded ecosystems and consequently to achieve multiple sustainable targets (Crowther et al. 2024). This can provide information for improving decontamination and restoration of contaminated sediments and to develop adaptive management strategies tailored to specific polluted conditions.

In conclusion, our results based on the genetic imprints preserved in the extracellular DNA pools allow us to provide new insights into responses of prokaryotic diversity potentially induced by natural and anthropogenic disturbance events. At the same time, the analysis of extracellular DNA pools, acting as a historical archive of genetic information, paves the way for improving our understanding of microbial succession over time and the related consequences on key ecosystem processes.

## Author Contributions

S.V. wrote the manuscript; A.D., C.C., L.M., R.D.: conceptualisation. S.V. and M.T.: data curation. S.V., M.T., A.S., G.A.: methodology and formal analysis. A.D., R.D., M.T., C.C., L.M., G.A., A.S.: review and editing. R.D., C.C., A.D.: funding acquisition. All authors have read and agreed to the published version of the manuscript.

## Conflicts of Interest

The authors declare no conflicts of interest.

## Supporting information


**Figure S1:** Location of the study area. The Tyrrhenian Sea (left) with a detailed view of the sampling sites in the Gulf of Naples (right). Core 1 was sampled at 40°48.150′ N, 14°08.913′ E, whereas Core 2 was sampled at 40°48.198′ N, 14°07.157′ E.
**Figure S2:** Overview of the analytical workflow applied to selected layers (highlighted in the dashed rectangle) of two sediment cores collected from the Bagnoli‐Coroglio Bay. Core 1 was useful to assess the impact of industrialisation (1911–1992) on the microbial communities, whereas the analyses of core 2 were useful to understand the microbial responses during an intensive volcanic activity. Sediment dating and chemical characterisation, combined with molecular analyses (qPCR and 16S rRNA metabarcoding), were carried out to assess changes in prokaryotic abundance and diversity over time. Metabarcoding data were useful for identifying potential resistant, sensitive and resilient prokaryotic taxa.
**Figure S3:** TapeStation 4200 profiles of extracellular DNA isolated from different sediment layers of two cores, analysed in three separate runs using ScreenTape D1000. Digital electrophoresis images show individual replicates from Core 1 (in red) and Core 2 (in blue) (A–C). The first lane in each gel image represents the DNA ladder ranging from 25 bp to 1500 bp (L). In D is reported a typical extracellular DNA fragment size distribution isolated from analysed samples, using the profile of nucleic acids isolated from the layer dated to 1726 as an example. The labels R1 and R2 indicate two independent DNA extraction replicates performed on each analysed sediment layer.
**Figure S4:** Rarefaction curves of ASVs obtained from the analysis of metabarcoding of 16S V4 rRNA amplified from extracellular DNA isolated from dated cores grouped in the legend with different colours according to the period identified. Rarefaction curves were generated for each sample after subsampling to a depth of 10,100 sequences.
**Figure S5:** Taxa barplot showing the relative abundances of the 20 most abundant prokaryotic classes over time in the core 1 and core 2. The labels R1 and R2 indicate two replicates of 16S rDNA metabarcoding performed on independent DNA extractions from each sediment layer analysed in this study.
**Figure S6:** Venn diagrams showing the number of ASVs shared between sediment cores collected during three distinct historical periods: Pre‐industrial period (1770–1785), Industrial period (1930–1940) and post‐industrial period (2013–2015). For each period, the diagrams illustrate the number of ASVs detected exclusively in Core 1 (green circle), exclusively in Core 2 (orange circle), and those shared between both cores (overlapping area). Percentages represent the proportion of reads attributed to each group within the total reads for that period.
**Table S1:** Estimation of years of sediment layers analysed within two cores and metal concentrations reported as mg kg^−1^. The column labelled as layer reported the names of samples used for 16S rDNA metabarcoding analyses.
**Table S2:** Concentrations of organic pollutants are reported as μg kg^−1^ for each sediment layer analysed.
**Table S3:** Results of ANOVA main tests of Bacterial and Archaeal gene abundances tested in two different cores over time.
**Table S4:** Pairwise comparisons test carried out on archaeal abundances (expressed as 16S rDNA copies) determined in the different sediment layers investigated within the two cores.
**Table S5:** Pairwise comparisons test carried out on bacterial abundances (expressed as 16S rDNA copies) determined in the different sediment layers investigated within the two cores.
**Table S6:** Results of *t*‐test comparisons between similar dated sediment layers from the analysed cores
**Table S7:** Number of reads, filtered, denoised and merged from sequencing of extracellular DNA samples isolated from sediment cores.
**Table S8:** Alpha‐diversity metrics of prokaryotic assemblages across the sediment layers of Core 1 and Core 2. Shannon diversity index (Shannon), Simpson diversity index (Simpson) and Pielou's evenness (Pielou) for each sample, grouped by core and corresponding historical period, are reported.
**Table S9:** Richness of ASV types across the core 1.
**Table S10:** Richness of ASV types across the core 2.
**Table S11:** Results of the PERMANOVA test assessing the comparisons of different periods (pre‐industrial, industrial, and post‐industrial) identified in the two cores. Significant differences are indicated by * for *p* < 0.05 and ** for *p* < 0.01.
**Table S12:** Results of differential abundance analysis (*emmeans_test*) of prokaryotic read abundances across different periods in core 1 (I1: pre‐industrial, I2: industrial, I3: post‐industrial) and core 2 (E1: pre‐eruption, E2: eruption, E3: post‐eruption). Mean relative abundances calculated for each period, along with standard deviation (SD) and standard error (SE), are reported. Different letters (a, b) indicate statistically significant differences between periods (*p* < 0.05).
**Table S13:** Results of the Mantel test assessing correlation (using Pearson's method) between prokaryotic gene copy numbers and different variables (Year, mERMq of polycyclic aromatic hydrocarbons (PAHs) and metals, and Bacterial and Archaeal 16S rRNA gene copy number (GCN)). The table shows the correlation coefficients (Mantel's *r*) and significance values (*p*‐values) for the Mantel test. Significant differences are indicated by * for *p* < 0.05 and ** for *p* < 0.01.
**Table S14:** Results of the differential abundance analysis of ASVs classified at the family level carried out by the *emmeans_test* procedure. The table reports the top‐ and bottom‐10 families with either the highest absolute values of the estimated mean differences in relative abundance, together with the estimate of the effect size (estimate) of differences between abundances of taxa identified in core1 and core2, standard errors (se), lower and upper bounds of the 95% confidence interval (conf low and conf high), the t.ratio test statistic (statistic), adjusted *p*‐values (*p*‐adjusted), significance levels (significance; values correspond to: * for 0.05, ** for 0.01, *** for 0.001 and **** for lower *p*‐values) and the corresponding periods: post‐industrial (2013–2015), industrial (1930–1940) and pre‐industrial (1770–1785).

## Data Availability

Sequences have been deposited in the NCBI's Sequence Read Archive under BioProject ID PRJNA1225128.
