# Supplementary material for: Decoding Past Microbial Communities Shifts Induced by Natural and Anthropogenic Disturbance Events Through Extracellular DNA
Source: Mol Ecol. 2025 Aug 22;34(20):e70078. doi: 10.1111/mec.70078 (PMC12530286; doi:10.1111/mec.70078)
Supplement: Supplementary file 1 — Figure S1: Location of the study area. The Tyrrhenian Sea (left) with a detailed view of the sampling sites in the Gulf of Naples (right). Core 1 was sampled at 40°48.150′ N, 14°08.913′ E, whereas Core 2 was sampled at 40°48.198′ N, 14°07.157′ E. Figure S2: Overview of the analytical workflow applied to selected layers (highlighted in the dashed rectangle) of two sediment cores collected from the Bagnoli‐Coroglio Bay. Core 1 was useful to assess the impact of industrialisation (1911–1992) on the microbial communities, whereas the analyses of core 2 were useful to understand the microbial responses during an intensive volcanic activity. Sediment dating and chemical characterisation, combined with molecular analyses (qPCR and 16S rRNA metabarcoding), were carried out to assess changes in prokaryotic abundance and diversity over time. Metabarcoding data were useful for identifying potential resistant, sensitive and resilient prokaryotic taxa. Figure S3: TapeStation 4200 profiles of extracellular DNA isolated from different sediment layers of two cores, analysed in three separate runs using ScreenTape D1000. Digital electrophoresis images show individual replicates from Core 1 (in red) and Core 2 (in blue) (A–C). The first lane in each gel image represents the DNA ladder ranging from 25 bp to 1500 bp (L). In D is reported a typical extracellular DNA fragment size distribution isolated from analysed samples, using the profile of nucleic acids isolated from the layer dated to 1726 as an example. The labels R1 and R2 indicate two independent DNA extraction replicates performed on each analysed sediment layer. Figure S4: Rarefaction curves of ASVs obtained from the analysis of metabarcoding of 16S V4 rRNA amplified from extracellular DNA isolated from dated cores grouped in the legend with different colours according to the period identified. Rarefaction curves were generated for each sample after subsampling to a depth of 10,100 sequences. Figure S5: Taxa barplot showing t [file MEC-34-e70078-s001.zip › 3_MEC_Supplemental_Information Varrella et alii MEC70078..docx]

**Supplemental Information for:**

**Manuscript Decoding past microbial communities shifts induced by natural and anthropogenic disturbance events through extracellular DNA**

Varrella S.^1,2^, Tangherlini M.^3^, Corinaldesi C.^2,4^, Musco L.^2,5^, Schirone A.^6^, Armiento G.^7^, Danovaro R.^1,2^, Dell’Anno A.^1,2^

*^1^Department of Life and Environmental Sciences, Polytechnic University of Marche, UNIVPM, Ancona, Italy;*

*^2^National Biodiversity Future Centre, Palermo, Italy;*

*^3^Stazione Zoologica Anton Dohrn, Naples, Italy;*

*^4^Department of Materials, Environmental Sciences and Urban Planning, UNIVPM, Ancona, Italy;*

*^5^Department of Biological and Environmental Sciences and Technologies, Salento University, Lecce, Italy;*

*^6^Department for Sustainability, ENEA Santa Teresa Research Centre, Località Pozzuolo di Lerici, Italy;*

*^7^Department for Sustainability, ENEA Casaccia Research Centre, Roma, Italy*

*Correspondence:*

*S. Varrella (s.varrella@univpm.it)*

**Supplemental figures**

**
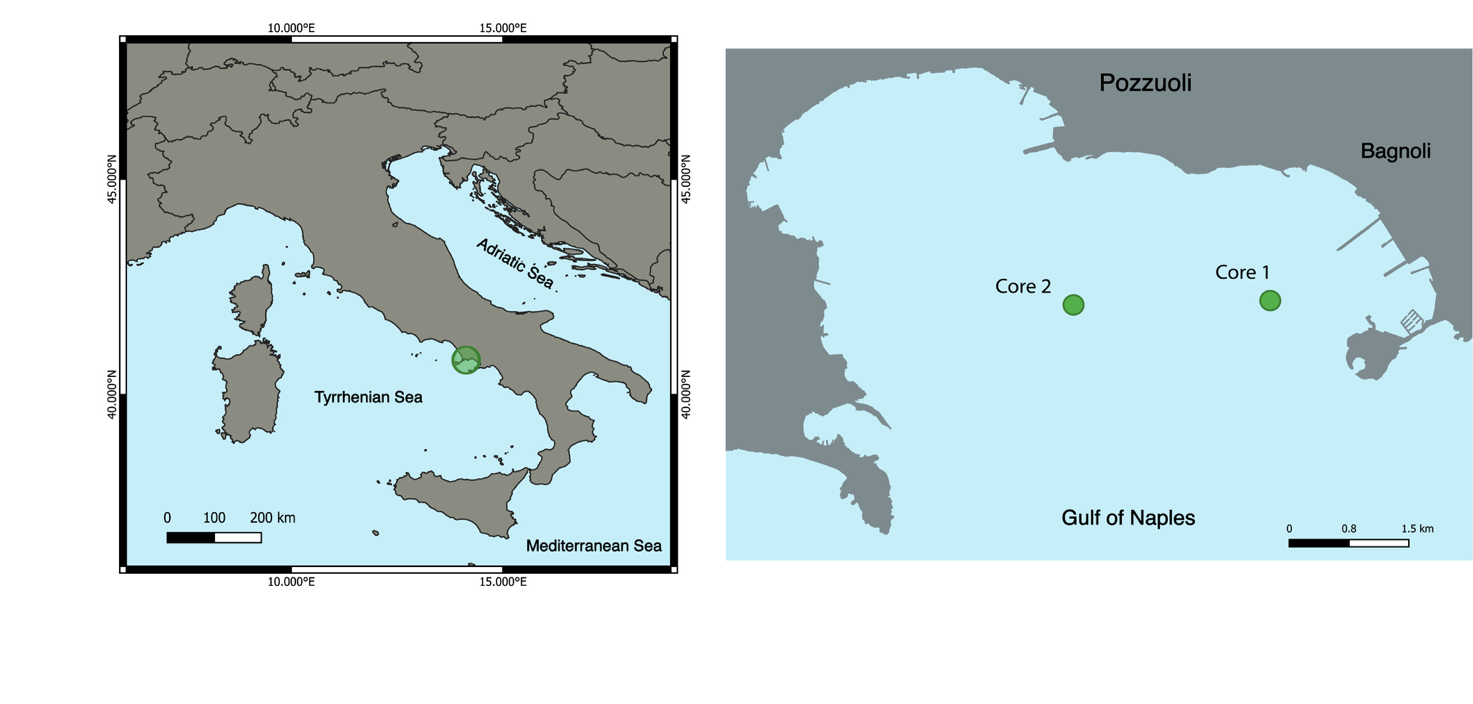
**

**Figure S1**. Location of the study area. The Tyrrhenian Sea (left) with a detailed view of the sampling sites in the Gulf of Naples (right). Core 1 was sampled at 40° 48.150’ N, 14° 08.913’ E, whereas Core 2 was sampled at 40° 48.198’ N, 14° 07.157’ E.


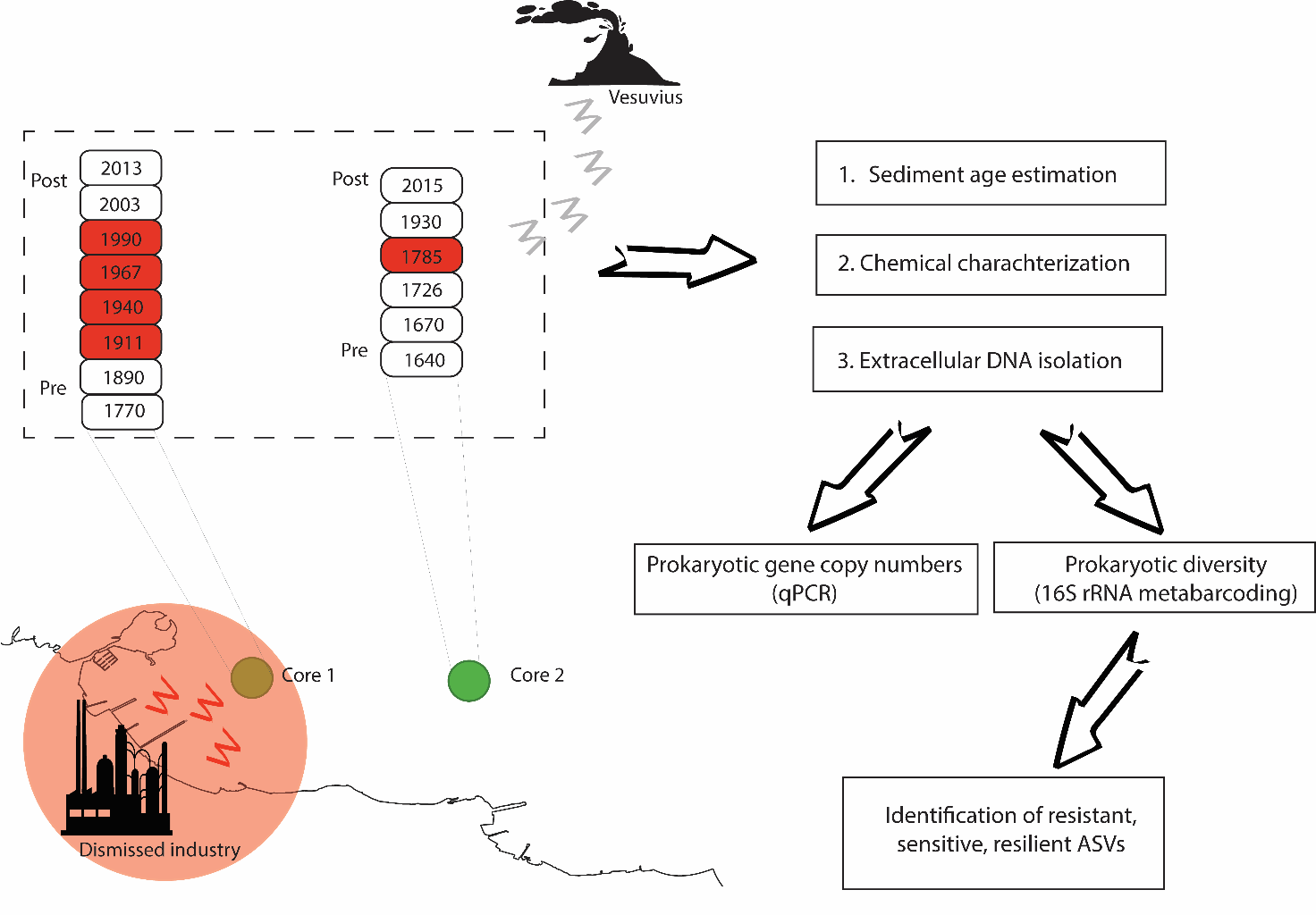


**Figure S2.** Overview of the analytical workflow applied to selected layers (highlighted in the dashed rectangle) of two sediment cores collected from the Bagnoli-Coroglio Bay. Core 1 was useful to assess the impact of industrialization (1911-1992) on the microbial communities, whereas the analyses of core 2 were useful to understand the microbial responses during an intensive volcanic activity. Sediment dating and chemical characterization, combined with molecular analyses (qPCR and 16S rRNA metabarcoding), were carried out to assess changes in prokaryotic abundance and diversity over time. Metabarcoding data were useful for identifying resistant, sensitive, and resilient prokaryotic taxa.


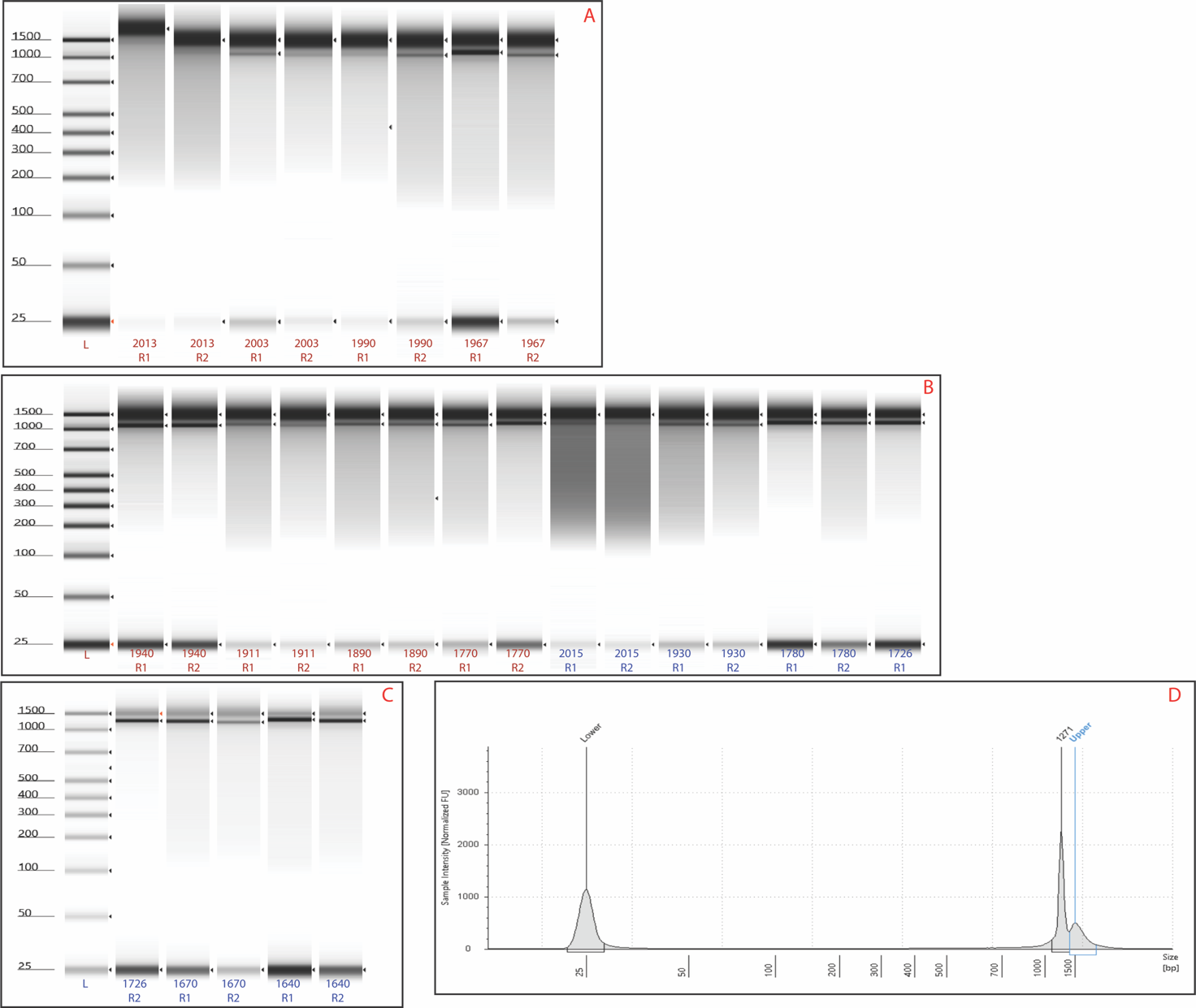


**Figure S3.** TapeStation 4200 profiles of extracellular DNA isolated from different sediment layers of two cores, analyzed in three separate runs using ScreenTape D1000. Digital electrophoresis images show individual replicates from Core 1 (in red) and Core 2 (in blue) (A–C). The first lane in each gel image represents the DNA ladder ranging from 25 bp to 1500 bp (L). In D is reported a typical extracellular DNA fragment size distribution isolated from analyzed samples, using the profile of nucleic acids isolated from the layer dated to 1726 as an example. The labels R1 and R2 indicate two independent DNA extraction replicates performed on each analysed sediment layer.


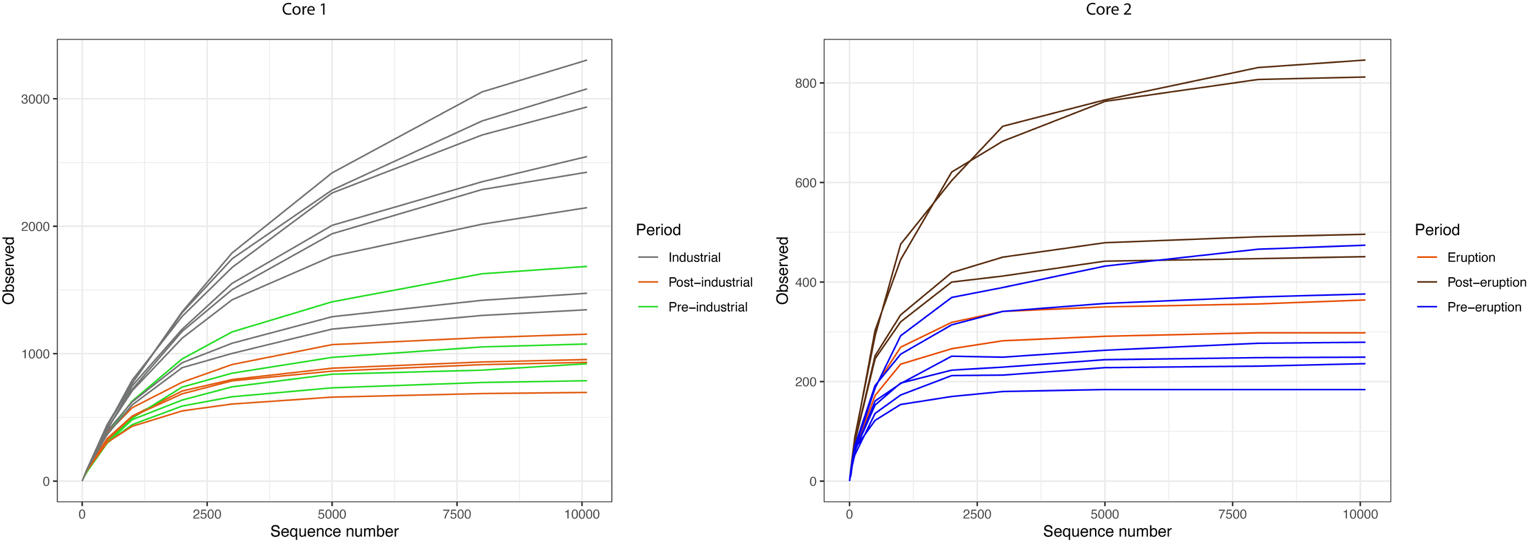


**Figure S4.** Rarefaction curves of ASVs obtained from the analysis of metabarcoding of 16S V4 rRNA amplified from extracellular DNA isolated from dated cores grouped in the legend with different colors according to the period identified. Rarefaction curves were generated for each sample after subsampling to a depth of 10,100 sequences.


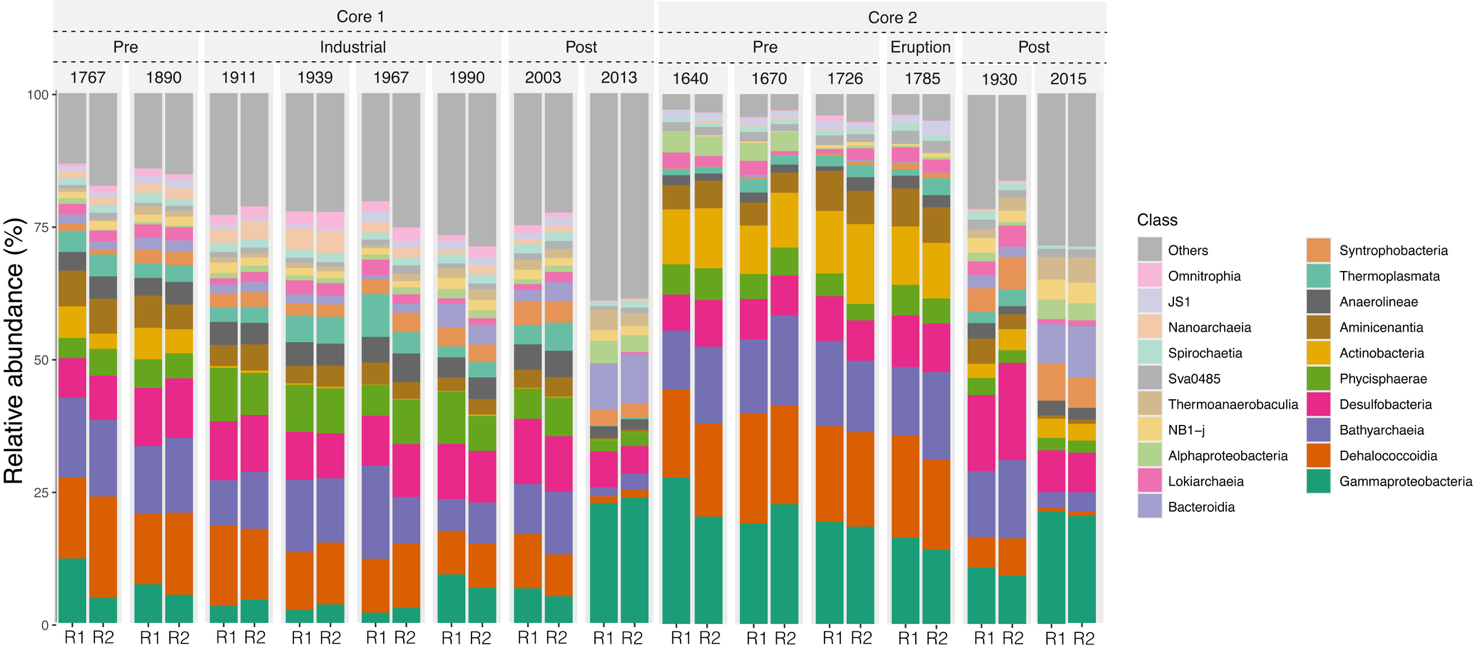


**Figure S5.** Taxa barplot showing the relative abundances of the 20 most abundant prokaryotic classes over time in the core 1 and core 2. The labels R1 and R2 indicate two replicates of 16S rDNA metabarcoding performed on independent DNA extractions from each sediment layer analyzed in this study.

**
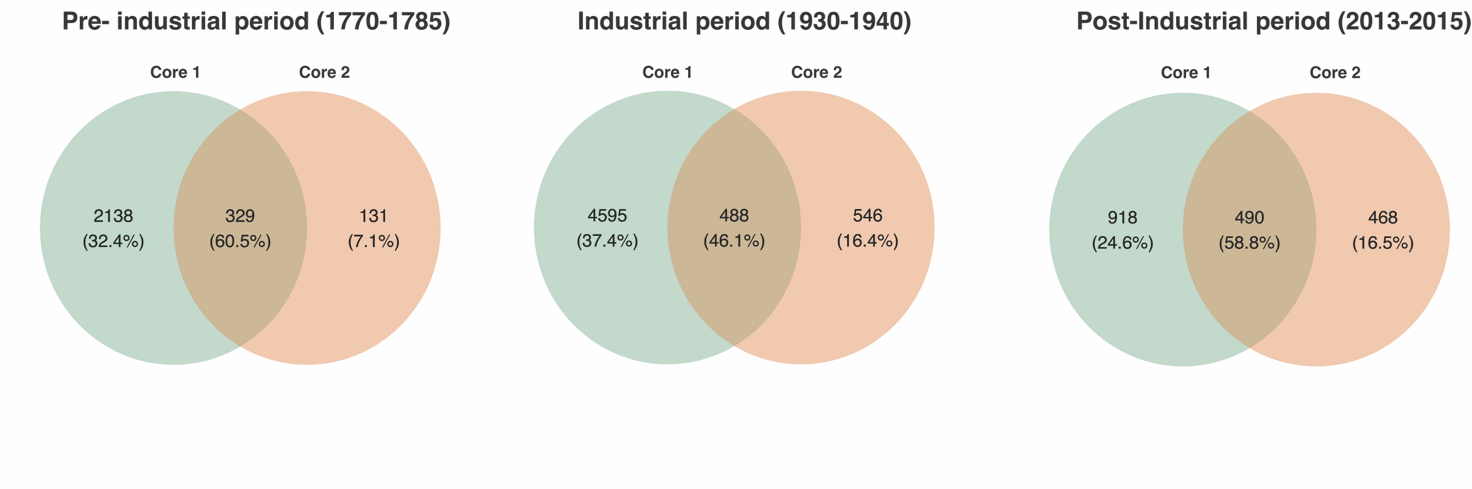
Figure S6.** Venn diagrams showing the number of ASVs shared between sediment cores collected during three distinct historical periods: Pre-industrial period (1770–1785), Industrial period (1930–1940), and Post-industrial period (2013–2015). For each period, the diagrams illustrate the number of ASVs detected exclusively in Core 1 (green circle), exclusively in Core 2 (orange circle), and those shared between both cores (overlapping area). Percentages represent the proportion of reads attributed to each group within the total reads for that period.

**Supplementary tables**

**Table S1.** Estimation of years of sediment layers analysed within two cores and metal concentrations reported as mg kg^-1^. The column labelled as layer reported the names of samples used for 16S rDNA metabarcoding analyses.

|  | **Layer** | **Year** | **Layer** | **Arsenic** | **Cadmium** | **Chromium** | **Copper** | **Mercury** | **Nickel** | **Lead** | **Zinc** | **mERMq** |
| --- | --- | --- | --- | --- | --- | --- | --- | --- | --- | --- | --- | --- |
| Core 1 | 1B1 | 2013 | 0-1 cm | 38.45 | 0.89 | 45.6 | 69.2 | 1.405 | 13.45 | 352 | 590.5 | 0.8 |
|  | 1B3 | 2003 | 5-6 cm | 41.75 | 1.55 | 41.35 | 85.35 | 1.97 | 14.35 | 426.5 | 758 | 1.00 |
|  | 1B5 | 1990 | 9-10 cm | 46.3 | 1.89 | 43.6 | 89.2 | 1.985 | 15.05 | 490 | 890 | 1.10 |
|  | 1B7 | 1967 | 13-14 cm | 51.3 | 2.525 | 50.1 | 111.5 | 2.57 | 19.2 | 599.5 | 1160 | 1.39 |
|  | 1B9 | 1940 | 17-18 cm | 41.6 | 1.51 | 31.9 | 106.5 | 2.64 | 12.9 | 395.5 | 808.5 | 1.12 |
|  | 1B12 | 1911 | 23-24 cm | 24.35 | 0.425 | 17.85 | 49.8 | 1.05 | 11.55 | 142.5 | 196 | 0.43 |
|  | 1B14 | 1890 | 27-28 cm | 24.5 | 0.36 | 16.25 | 40.15 | 0.875 | 10.2 | 125 | 163 | 0.37 |
|  | 1B27 | 1770 | 53-54 cm | 24.2 | 0.2 | 15.35 | 21.9 | 0.355 | 10.5 | 73.6 | 74.3 | 0.21 |
| Core 2 | 2B1 | 2015 | 0-1 cm | 28.75 | 0.285 | 38.3 | 26.35 | 0.575 | 13.6 | 139.5 | 206.5 | 0.35 |
|  | 2B8 | 1930 | 15-16 cm | 24.7 | 0.245 | 24.8 | 25.8 | 0.47 | 12.65 | 95.1 | 114 | 0.27 |
|  | 2B15 | 1785 | 29-30 cm | 25.15 | 0.22 | 20.15 | 16.35 | 0.135 | 12.1 | 56.9 | 69.95 | 0.17 |
|  | 2B18 | 1726 | 35-36 cm | 25.05 | 0.19 | 20.35 | 17.65 | 0.08 | 11.95 | 55.5 | 67.65 | 0.15 |
|  | 2B21 | 1670 | 41-42 cm | 24.55 | 0.19 | 19.4 | 17.75 | 0.13 | 12 | 59.7 | 67.3 | 0.17 |
|  | 2B24 | 1640 | 47-48 cm | 22.9 | 0.21 | 16.6 | 20.9 | 0.245 | 10.45 | 68.75 | 68.2 | 0.19 |

**Table S2.** Concentrations of organic pollutants are reported as μg kg^-1^ for each sediment layer analysed.

|  | **Year** | **Naphtalene** | **Acenaphthylene** | **Acenaphthene** | **Fluorene** | **Phenanthrene** | **Anthracene** | **Fluoranthene** | **Pyrene** | **B(a)anthracene** | **Chrysene** | **B(a)pyrene** | **Dib(ah)antracene** | **Sum of 16 PAHs** | **mERMq** |
| --- | --- | --- | --- | --- | --- | --- | --- | --- | --- | --- | --- | --- | --- | --- | --- |
| Core 1 | 2013 | 1495 | 569.5 | 723 | 871.5 | 7975.5 | 3831 | 26704 | 19692.5 | 13998 | 11329.5 | 26676 | 6257.5 | 198291 | 6.47 |
|  | 2003 | 1473 | 603.5 | 630.5 | 1162 | 9372 | 4160.5 | 23703.5 | 18960.5 | 13008 | 10727.5 | 26108.5 | 6216 | 191039.5 | 6.42 |
|  | 1990 | 1601.5 | 529.5 | 655 | 699 | 5698.5 | 2841.5 | 19434 | 15538.5 | 14166.5 | 11732 | 23378.5 | 5591 | 165310.5 | 5.63 |
|  | 1967 | 2988 | 1018.5 | 1140 | 1229 | 9645 | 4811.5 | 31821.5 | 24036 | 31950.5 | 23817 | 37968 | 6523.5 | 256273 | 8.99 |
|  | 1940 | 618 | 209.5 | 138.5 | 251.5 | 2195.5 | 1251 | 7571.5 | 5711 | 5185 | 4713.5 | 9858 | 2733.5 | 67534.5 | 2.36 |
|  | 1911 | 56 | 15.5 | 16.5 | 32.5 | 333 | 188.5 | 1008 | 761.5 | 852 | 836 | 1077 | 256.5 | 8611.5 | 0.28 |
|  | 1890 | 49 | 10.5 | 0 | 20 | 270 | 134 | 768.5 | 581 | 608 | 626.5 | 679 | 30.5 | 5380 | 0.15 |
|  | 1770 | 16.5 | 0.5 | 9 | 5.5 | 67.5 | 7 | 71 | 63.5 | 85.5 | 54.5 | 67.5 | 17 | 665 | 0.02 |
| Core 2 | 2015 | 271.5 | 141.5 | 63.5 | 139 | 1892 | 733 | 5984 | 4423 | 2384 | 2052 | 4421.5 | 1059.5 | 37805.5 | 1.18 |
|  | 1930 | 74.5 | 43 | 11.5 | 34.5 | 430.5 | 186 | 1384.5 | 978 | 707.5 | 657.5 | 1295.5 | 191.5 | 9406 | 0.28 |
|  | 1785 | 6 | 4 | 2 | 4 | 38 | 12 | 53 | 46 | 31 | 36 | 60 | 8 | 506 | 0.01 |
|  | 1726 | 8 | 3.5 | 2 | 4 | 40.5 | 14.5 | 74.5 | 57 | 21.5 | 30.5 | 35.5 | 4 | 435 | 0.01 |
|  | 1670 | 8.5 | 3 | 1.5 | 4 | 50 | 15.5 | 105 | 73.5 | 22 | 35 | 20 | 2.5 | 450.5 | 0.01 |
|  | 1640 | 7 | 3 | 1 | 4 | 57 | 14 | 114 | 79 | 32 | 45 | 29 | 5 | 537 | 0.01 |

**Table S3.** Results of ANOVA main tests of Bacterial and Archaeal gene abundances tested in two different cores over time.

|  | **Bacteria** | | **Archaea** | |
| --- | --- | --- | --- | --- |
|  | **Core 1** | **Core 2** | **Core 1** | **Core 2** |
| F | 2.311 | 8.744 | 62.72 | 5.659 |
| p-value | 0.0446 | <0.0001 | <0.0001 | 0.0009 |
| R squared | 0.288 | 0.5931 | 0.9165 | 0.4854 |

**Table S4.** Pairwise comparisons test carried out on archaeal abundances (expressed as 16S rDNA copies) determined in the different sediment layers investigated within the two cores. Significance values correspond to: * for 0.05, ** for 0.01, *** for 0.001 and **** for lower p-values.

|  | **Tukey's multiple comparisons test** | **Mean Diff** | **95.00% CI of diff** | **Summary** | **Adjusted P Value** |
| --- | --- | --- | --- | --- | --- |
| Core 1 | 1770 vs. 1890 | 29810000 | 14203595 to 45416405 | **** | <0.0001 |
|  | 1770 vs. 1911 | 19150000 | 3543595 to 34756405 | ** | 0.0074 |
|  | 1770 vs. 1940 | -43333333 | -58939738 to -27726928 | **** | <0.0001 |
|  | 1770 vs. 1967 | 28883333 | 13276928 to 44489738 | **** | <0.0001 |
|  | 1770 vs. 1990 | 33695000 | 18088595 to 49301405 | **** | <0.0001 |
|  | 1770 vs. 2003 | 32715000 | 17108595 to 48321405 | **** | <0.0001 |
|  | 1770 vs. 2013 | 38671667 | 23065262 to 54278072 | **** | <0.0001 |
|  | 1890 vs. 1911 | -10660000 | -26266405 to 4946405 | ns | 0.3829 |
|  | 1890 vs. 1930 | -73143333 | -88749738 to -57536928 | **** | <0.0001 |
|  | 1890 vs. 1967 | -926667 | -16533072 to 14679738 | ns | >0.9999 |
|  | 1890 vs. 1990 | 3885000 | -11721405 to 19491405 | ns | 0.9924 |
|  | 1890 vs. 2003 | 2905000 | -12701405 to 18511405 | ns | 0.9988 |
|  | 1890 vs. 2013 | 8861667 | -6744738 to 24468072 | ns | 0.614 |
|  | 1911 vs. 1940 | -62483333 | -78089738 to -46876928 | **** | <0.0001 |
|  | 1911 vs. 1967 | 9733333 | -5873072 to 25339738 | ns | 0.4987 |
|  | 1911 vs. 1990 | 14545000 | -1061405 to 30151405 | ns | 0.0834 |
|  | 1911 vs. 2003 | 13565000 | -2041405 to 29171405 | ns | 0.1295 |
|  | 1911 vs. 2013 | 19521667 | 3915262 to 35128072 | ** | 0.0059 |
|  | 1940 vs. 1967 | 72216667 | 56610262 to 87823072 | **** | <0.0001 |
|  | 1940 vs. 1990 | 77028333 | 61421928 to 92634738 | **** | <0.0001 |
|  | 1940 vs. 2003 | 76048333 | 60441928 to 91654738 | **** | <0.0001 |
|  | 1940 vs. 2013 | 82005000 | 66398595 to 97611405 | **** | <0.0001 |
|  | 1967 vs. 1990 | 4811667 | -10794738 to 20418072 | ns | 0.974 |
|  | 1967 vs. 2003 | 3831667 | -11774738 to 19438072 | ns | 0.993 |
|  | 1967 vs. 2013 | 9788333 | -5818072 to 25394738 | ns | 0.4915 |
|  | 1990 vs. 2003 | -980000 | -16586405 to 14626405 | ns | >0.9999 |
|  | 1990 vs. 2013 | 4976667 | -10629738 to 20583072 | ns | 0.9687 |
|  | 2003 vs. 2013 | 5956667 | -9649738 to 21563072 | ns | 0.9211 |
| Core 2 | 1640 vs. 1670 | 715000 | -16054133 to 17484133 | ns | >0.9999 |
|  | 1640 vs. 1726 | -10135000 | -24657495 to 4387495 | ns | 0.3103 |
|  | 1640 vs. 1785 | -20088333 | -36857466 to -3319201 | * | 0.0112 |
|  | 1640 vs. 1930 | -7761667 | -24530799 to 9007466 | ns | 0.7311 |
|  | 1640 vs. 2015 | 1412167 | -15356966 to 18181299 | ns | 0.9998 |
|  | 1670 vs. 1726 | -10850000 | -25372495 to 3672495 | ns | 0.2419 |
|  | 1670 vs. 1785 | -20803333 | -37572466 to -4034201 | ** | 0.0079 |
|  | 1670 vs. 1930 | -8476667 | -25245799 to 8292466 | ns | 0.6535 |
|  | 1670 vs. 2015 | 697167 | -16071966 to 17466299 | ns | >0.9999 |
|  | 1726 vs. 1785 | -9953333 | -24475828 to 4569162 | ns | 0.3293 |
|  | 1726 vs. 1930 | 2373333 | -12149162 to 16895828 | ns | 0.9962 |
|  | 1726 vs. 2015 | 11547167 | -2975328 to 26069662 | ns | 0.1860 |
|  | 1785 vs. 1930 | 12326667 | -4442466 to 29095799 | ns | 0.2576 |
|  | 1785 vs. 2015 | 21500500 | 4731367 to 38269633 | ** | 0.0056 |
|  | 1930 vs. 2015 | 9173833 | -7595299 to 25942966 | ns | 0.5748 |

**Table S5.** Pairwise comparisons test carried out on bacterial abundances ((expressed as 16S rDNA copies) determined in the different sediment layers investigated within the two cores. Significance values correspond to: * for 0.05, ** for 0.01, *** for 0.001 and **** for lower p-values.

|  | **Tukey's multiple comparisons test** | **Mean Diff.** | **95,00% CI of diff** | **Summary** | **Adjusted P Value** |
| --- | --- | --- | --- | --- | --- |
| **Core 1** | 1770 vs. 1890 | 2120000 | -509330 to 4749330 | ns | 0.1943 |
|  | 1770 vs. 1911 | 2443333 | -185997 to 5072663 | ns | 0.0851 |
|  | 1770 vs. 1940 | 438333 | -2190997 to 3067663 | ns | 0.9994 |
|  | 1770 vs. 1967 | 1088333 | -1540997 to 3717663 | ns | 0.8846 |
|  | 1770 vs. 1990 | 1986667 | -642663 to 4615997 | ns | 0.2621 |
|  | 1770 vs. 2003 | 1920000 | -709330 to 4549330 | ns | 0.3012 |
|  | 1770 vs. 2013 | 1988333 | -640997 to 4617663 | ns | 0.2611 |
|  | 1890 vs. 1911 | 323333 | -2305997 to 2952663 | ns | >0.9999 |
|  | 1890 vs. 1930 | -1681667 | -4310997 to 947663 | ns | 0.4666 |
|  | 1890 vs. 1967 | -1031667 | -3660997 to 1597663 | ns | 0.9099 |
|  | 1890 vs. 1990 | -133333 | -2762663 to 2495997 | ns | >0.9999 |
|  | 1890 vs. 2003 | -200000 | -2829330 to 2429330 | ns | >0.9999 |
|  | 1890 vs. 2013 | -131667 | -2760997 to 2497663 | ns | >0.9999 |
|  | 1911 vs. 1940 | -2005000 | -4634330 to 624330 | ns | 0.2519 |
|  | 1911 vs. 1967 | -1355000 | -3984330 to 1274330 | ns | 0.7195 |
|  | 1911 vs. 1990 | -456667 | -3085997 to 2172663 | ns | 0.9992 |
|  | 1911 vs. 2003 | -523333 | -3152663 to 2105997 | ns | 0.9981 |
|  | 1911 vs. 2013 | -455000 | -3084330 to 2174330 | ns | 0.9992 |
|  | 1940 vs. 1967 | 650000 | -1979330 to 3279330 | ns | 0.9927 |
|  | 1940 vs. 1990 | 1548333 | -1080997 to 4177663 | ns | 0.5704 |
|  | 1940 vs. 2003 | 1481667 | -1147663 to 4110997 | ns | 0.6229 |
|  | 1940 vs. 2013 | 1550000 | -1079330 to 4179330 | ns | 0.5691 |
|  | 1967 vs. 1990 | 898333 | -1730997 to 3527663 | ns | 0.9548 |
|  | 1967 vs. 2003 | 831667 | -1797663 to 3460997 | ns | 0.97 |
|  | 1967 vs. 2013 | 900000 | -1729330 to 3529330 | ns | 0.9544 |
|  | 1990 vs. 2003 | -66667 | -2695997 to 2562663 | ns | >0.9999 |
|  | 1990 vs. 2013 | 1667 | -2627663 to 2630997 | ns | >0.9999 |
|  | 2003 vs. 2013 | 68333 | -2560997 to 2697663 | ns | >0.9999 |
| Core 2 | 1640 vs. 1670 | -430667 | -3589209 to 2727876 | ns | 0.9983 |
|  | 1640 vs. 1726 | -4175667 | -7334209 to -1017124 | ** | 0.0044 |
|  | 1640 vs. 1785 | -5099000 | -8257543 to -1940457 | *** | 0.0004 |
|  | 1640 vs. 1930 | -4494000 | -7652543 to -1335457 | ** | 0.002 |
|  | 1640 vs. 2015 | -3152333 | -6310876 to 6209 | ns | 0.0507 |
|  | 1670 vs. 1726 | -3745000 | -6903543 to -586457 | * | 0.0129 |
|  | 1670 vs. 1785 | -4668333 | -7826876 to -1509791 | ** | 0.0012 |
|  | 1670 vs. 1930 | -4063333 | -7221876 to -904791 | ** | 0.0059 |
|  | 1670 vs. 2015 | -2721667 | -5880209 to 436876 | ns | 0.1232 |
|  | 1726 vs. 1785 | -923333 | -4081876 to 2235209 | ns | 0.9462 |
|  | 1726 vs. 1930 | -318333 | -3476876 to 2840209 | ns | 0.9996 |
|  | 1726 vs. 2015 | 1023333 | -2135209 to 4181876 | ns | 0.919 |
|  | 1785 vs. 1930 | 605000 | -2553543 to 3763543 | ns | 0.9915 |
|  | 1785 vs. 2015 | 1946667 | -1211876 to 5105209 | ns | 0.4364 |
|  | 1930 vs. 2015 | 1341667 | -1816876 to 4500209 | ns | 0.787 |

**Table S6.** Results of t-test comparisons between similar dated sediment layers from the analyzed cores

|  | **Archaea** | | |
| --- | --- | --- | --- |
|  | **1770vs1785** | **1930vs1940** | **2013vs2015** |
| p-value | \| 0.036916 \| \| --- \| | <0.0001 | <0.0001 |
| t ratio | 2.406 | 15.81 | 7.162 |
| q value | 0.012428 | <0.000001 | 0.000015 |
|  | **Bacteria** | | |
|  | **1770vs1785** | **1930vs1940** | **2013vs2015** |
| p-value | 0.405472 | 0.358319 | 0.130565 |
| t ratio | 0.8685 | 0.9629 | 1.647 |
| q value | 0.409527 | 0.409527 | 0.395611 |

**Table S7**. Number of reads, filtered, denoised, and merged from sequencing of extracellular DNA samples isolated from sediment cores

| **Sample** | **Reads input** | **Filtered** | **Denoised** | **Merged** | **Resulted Reads** |
| --- | --- | --- | --- | --- | --- |
| 2013_r1 | 77891 | 67301 | 54221 | 34172 | 27065 |
| 2013_r2 | 63155 | 53165 | 41236 | 25128 | 19041 |
| 2003_r1 | 72418 | 63374 | 52073 | 34697 | 25518 |
| 2003_r2 | 88203 | 77459 | 65376 | 46708 | 33695 |
| 1990_r1 | 144100 | 126971 | 105759 | 64695 | 46382 |
| 1990_r2 | 679282 | 599228 | 572499 | 498889 | 361882 |
| 1967_r1 | 98297 | 88192 | 77477 | 59640 | 43798 |
| 1967_r2 | 492948 | 441517 | 420678 | 369037 | 265335 |
| 1940_r1 | 337704 | 300352 | 285681 | 248520 | 194325 |
| 1940_r2 | 164960 | 148708 | 136202 | 110009 | 87673 |
| 1911_r1 | 253070 | 227829 | 215411 | 186663 | 142585 |
| 1911_r2 | 219816 | 198676 | 186990 | 160224 | 127175 |
| 1890_r1 | 86056 | 74419 | 63861 | 45650 | 34080 |
| 1890_r2 | 62657 | 53858 | 45526 | 34017 | 25763 |
| 1770_r1 | 113917 | 99968 | 86993 | 63867 | 47231 |
| 1770_r2 | 163875 | 142439 | 129670 | 103863 | 82122 |
| 2015_r1 | 59108 | 52199 | 43522 | 30626 | 25209 |
| 2015_r2 | 116828 | 103066 | 90522 | 70211 | 56248 |
| 1930_r1 | 107721 | 94789 | 80561 | 57276 | 46319 |
| 1930_r2 | 60258 | 52964 | 41786 | 27320 | 21419 |
| 1785_r1 | 75559 | 63950 | 55461 | 40438 | 29658 |
| 1785_r2 | 87568 | 74258 | 66072 | 51941 | 38242 |
| 1726_r1 | 51174 | 43199 | 37120 | 27345 | 19360 |
| 1726_r2 | 181440 | 151182 | 141595 | 119072 | 84288 |
| 1670_r1 | 131611 | 109367 | 99454 | 77528 | 54998 |
| 1670_r2 | 78465 | 65320 | 58520 | 45265 | 31419 |
| 1640_r1 | 89729 | 77764 | 71596 | 59200 | 42781 |
| 1640_r2 | 75374 | 65341 | 59243 | 47794 | 34582 |

**Table S8.** Alpha diversity metrics of prokaryotic assemblages across the sediment layers of Core 1 and Core 2. Shannon diversity index (Shannon), Simpson diversity index (Simpson), and Pielou’s evenness (Pielou) for each sample, grouped by core and corresponding historical period, are reported.

| **Sample** | **Core** | **Period** | **Shannon** | **Simpson** | **Pielou** |
| --- | --- | --- | --- | --- | --- |
| 2013 R1 | Core 1 | Post-industrial | 6.539774 | 0.997947 | 0.946046 |
| 2013 R2 | Core 1 | Post-industrial | 6.17752 | 0.997133 | 0.938742 |
| 2003 R1 | Core 1 | Post-industrial | 6.444729 | 0.997391 | 0.937102 |
| 2003 R2 | Core 1 | Post-industrial | 6.701264 | 0.998005 | 0.942637 |
| 1990 R1 | Core 1 | Industrial | 6.952852 | 0.998386 | 0.943048 |
| 1990 R2 | Core 1 | Industrial | 7.914295 | 0.999074 | 0.94697 |
| 1967 R1 | Core 1 | Industrial | 6.85424 | 0.998305 | 0.940099 |
| 1967 R2 | Core 1 | Industrial | 7.781853 | 0.998855 | 0.942234 |
| 1940 R1 | Core 1 | Industrial | 7.761707 | 0.99902 | 0.94728 |
| 1940 R2 | Core 1 | Industrial | 7.399098 | 0.998912 | 0.94661 |
| 1911 R1 | Core 1 | Industrial | 7.543908 | 0.998755 | 0.939265 |
| 1911 R2 | Core 1 | Industrial | 7.47747 | 0.998517 | 0.937871 |
| 1890 R1 | Core 1 | Pre-industrial | 6.162554 | 0.992643 | 0.896611 |
| 1890 R2 | Core 1 | Pre-industrial | 6.131015 | 0.994788 | 0.914809 |
| 1770 R1 | Core 1 | Pre-industrial | 6.21185 | 0.988304 | 0.880558 |
| 1770 R2 | Core 1 | Pre-industrial | 6.98038 | 0.997216 | 0.924924 |
| 2015 R1 | Core 2 | Post-eruption | 5.735185 | 0.994018 | 0.922559 |
| 2015 R2 | Core 2 | Post-eruption | 6.161753 | 0.995166 | 0.914779 |
| 1930 R1 | Core 2 | Post-eruption | 6.259258 | 0.996308 | 0.924921 |
| 1930 R2 | Core 2 | Post-eruption | 5.585709 | 0.992464 | 0.910061 |
| 1785 R1 | Core 2 | Eruption | 4.748634 | 0.966555 | 0.833029 |
| 1785 R2 | Core 2 | Eruption | 4.964907 | 0.971906 | 0.841524 |
| 1726 R1 | Core 2 | Pre-eruption | 4.265977 | 0.952825 | 0.818031 |
| 1726 R2 | Core 2 | Pre-eruption | 4.783802 | 0.953419 | 0.771518 |
| 1670 R1 | Core 2 | Pre-eruption | 4.819383 | 0.962846 | 0.810962 |
| 1670 R2 | Core 2 | Pre-eruption | 4.294527 | 0.945091 | 0.785384 |
| 1640 R1 | Core 2 | Pre-eruption | 4.245854 | 0.930851 | 0.753507 |

**Table S9**. Richness of ASV types across the core 1.

| **Year** | **Total ASVs** | **Resistant** | **Resilient** | **Pre-industrial** | **Post-industrial** | **Industrial** | **Sensitive** | **Post-ind and industrial** |
| --- | --- | --- | --- | --- | --- | --- | --- | --- |
| 2013 | 1408 | 175 | 8 | 0 | 762 | 0 | 0 | 463 |
| 2003 | 1789 | 458 | 23 | 0 | 652 | 0 | 0 | 656 |
| 1990 | 5090 | 427 | 0 | 0 | 0 | 3426 | 498 | 739 |
| 1967 | 4622 | 398 | 0 | 0 | 0 | 3255 | 462 | 507 |
| 1940 | 5081 | 411 | 0 | 0 | 0 | 3716 | 582 | 372 |
| 1911 | 5048 | 380 | 0 | 0 | 0 | 3679 | 761 | 228 |
| 1890 | 1422 | 343 | 12 | 504 | 0 | 0 | 563 | 0 |
| 1770 | 2467 | 392 | 19 | 1226 | 0 | 0 | 830 | 0 |

**Table S10**. Richness of ASV types across the core 2

| **Year** | **Total ASVs** | **Resistant** | **Resilient** | **Pre-eruption** | **Post-eruption** | **Eruption** | **Sensitive** | **Post and during eruption** |
| --- | --- | --- | --- | --- | --- | --- | --- | --- |
| 2015 | 958 | 44 | 20 | 0 | 885 | 0 | 0 | 9 |
| 1930 | 1034 | 147 | 82 | 0 | 771 | 0 | 0 | 34 |
| 1785 | 460 | 149 | 0 | 0 | 0 | 74 | 201 | 36 |
| 1726 | 522 | 128 | 60 | 174 | 0 | 0 | 160 | 0 |
| 1670 | 437 | 119 | 37 | 129 | 0 | 0 | 152 | 0 |
| 1640 | 369 | 101 | 22 | 95 | 0 | 0 | 151 | 0 |

**Table S11.** Results of the PERMANOVA test assessing the comparisons of different periods (pre-industrial, industrial, and post-industrial) identified in the two cores. Significant differences are indicated by * for p < 0.05 and ** for p < 0.01.

| **Core** | **Groups** | **F** | **R^2^** | **Adjusted**  **p-value** | **significance** |
| --- | --- | --- | --- | --- | --- |
| Core 1 | Post-industrial vs Industrial | 2.09328 | 0.173095 | 0.012 | * |
|  | Post-industrial vs Pre-industrial | 3.119801 | 0.342091 | 0.034 | * |
|  | Industrial vs Pre-industrial | 2.65217 | 0.209622 | 0.006 | ** |
| Core 2 | Post-eruption vs eruption | 4.45278 | 0.526783 | 0.066667 |  |
|  | Post-eruption vs Pre-eruption | 12.70927 | 0.6137 | 0.006 | ** |
|  | Eruption vs Pre-eruption | 1.763746 | 0.227177 | 0.0525 |  |

**Table S13**. Results of the Mantel test assessing correlation (using Pearson’s method) between prokaryotic gene copy numbers and different variables (Year, mERMq of Polycyclic aromatic hydrocarbons (PAHs) and metals, and Bacterial and Archaeal 16S rRNA gene copy number (GCN)). The table shows the correlation coefficients (Mantel’s r) and significance values (p-values) for the Mantel test. Significant differences are indicated by * for p < 0.05 and ** for p < 0.01.

| **core** | **Variables** | **Correlation coefficient** | **p-value** | **p-adjusted** | **Significance** |
| --- | --- | --- | --- | --- | --- |
| 1 | Year | 0.567464 | 0.007 | 0.011 | * |
| 1 | mERMq_PAH | 0.48246 | 0.001 | 0.001833 | ** |
| 1 | mERMq _metals | 0.418991 | 0.001 | 0.001833 | ** |
| 1 | Bacterial_GCN_R1 | 0.648286 | 0.001 | 0.001833 | ** |
| 1 | Bacterial_GCN_R2 | 0.746106 | 0.001 | 0.001833 | ** |
| 1 | Bacterial_GCN_R3 | 0.752674 | 0.001 | 0.001833 | ** |
| 1 | Bacterial_GCN_avg | 0.729239 | 0.001 | 0.001833 | ** |
| 1 | Archaeal_GCN_R1 | -0.0685 | 0.594 | 0.726 |  |
| 1 | Archaeal_GCN_R2 | -0.00241 | 0.454 | 0.62425 |  |
| 1 | Archaeal_GCN_R3 | -0.09026 | 0.707 | 0.7777 |  |
| 1 | Archaeal_GCN_avg | -0.10974 | 0.81 | 0.81 |  |
| 2 | Year | 0.871008 | 0.001 | 0.001571 | ** |
| 2 | mERMq_PAH | 0.806764 | 0.001 | 0.001571 | ** |
| 2 | mERMq _metals | 0.92659 | 0.001 | 0.001571 | ** |
| 2 | Bacterial_GCN_R1 | 0.828533 | 0.001 | 0.001571 | ** |
| 2 | Bacterial_GCN_R2 | 0.876645 | 0.001 | 0.001571 | ** |
| 2 | Bacterial_GCN_R3 | 0.766628 | 0.001 | 0.001571 | ** |
| 2 | Bacterial_GCN_avg | 0.832049 | 0.001 | 0.001571 | ** |
| 2 | Archaeal_GCN_R1 | 0.355791 | 0.086 | 0.105111 |  |
| 2 | Archaeal_GCN_R2 | 0.219631 | 0.112 | 0.112 |  |
| 2 | Archaeal_GCN_R3 | 0.331378 | 0.053 | 0.072875 |  |
| 2 | Archaeal_GCN_avg | 0.311211 | 0.104 | 0.112 |  |

**Table S14.** Results of the differential abundance analysis of ASVs classified at the family level carried out by the *emmeans_test* procedure. The table reports the top- and bottom-10 families with either the highest absolute values of the estimated mean differences in relative abundance, together with the estimate of the effect size (estimate) of differences between abundance of taxa identified in core1 and core2, standard errors (se), lower and upper bounds of the 95% confidence interval (conf low and conf high), the t.ratio test statistic (statistic), adjusted p-values (p-adjusted), significance levels (significance; values correspond to: * for 0.05, ** for 0.01, *** for 0.001 and **** for lower p-values), and the corresponding periods: post-industrial (2013-2015), industrial (1930-1940) and pre-industrial (1770-1785).

| **Family** | **estimate** | **se** | **conf low** | **conf high** | **statistic** | **p-adjusted** | **significance** | **period** |
| --- | --- | --- | --- | --- | --- | --- | --- | --- |
| Subgroup_22 | 0.01891089 | 0.00142078 | 0.01612236 | 0.02169942 | 13.3102193 | 6.01E-37 | **** | post |
| Nitrosopumilaceae | 0.0179703 | 0.00142078 | 0.01518177 | 0.02075883 | 12.6481927 | 8.45E-34 | **** | post |
| Desulfobulbaceae | 0.01321782 | 0.00142078 | 0.01042929 | 0.01600635 | 9.30321609 | 1.06E-19 | **** | post |
| Woeseiaceae | 0.01034653 | 0.00142078 | 0.007558 | 0.01313506 | 7.28229274 | 7.31E-13 | **** | post |
| Thermodesulfovibrionia | 0.00742574 | 0.00142078 | 0.00463721 | 0.01021427 | 5.22652589 | 2.16E-07 | **** | post |
| Ectothiorhodospiraceae | 0.00678218 | 0.00142078 | 0.00399365 | 0.00957071 | 4.77356031 | 2.12E-06 | **** | post |
| BD7-8 | 0.00668317 | 0.00142078 | 0.00389464 | 0.0094717 | 4.7038733 | 2.96E-06 | **** | post |
| Pirellulaceae | 0.00633663 | 0.00142078 | 0.0035481 | 0.00912516 | 4.45996876 | 9.26E-06 | **** | post |
| Latescibacterota | 0.00623762 | 0.00142078 | 0.00344909 | 0.00902615 | 4.39028175 | 1.27E-05 | **** | post |
| BD2-11_terrestrial_group | 0.00589109 | 0.00142078 | 0.00310256 | 0.00867962 | 4.14637721 | 3.71E-05 | **** | post |
| Lokiarchaeia | -0.0076733 | 0.00142078 | -0.0104618 | -0.0048847 | -5.4007434 | 8.55E-08 | **** | post |
| Bathyarchaeia | -0.0085644 | 0.00142078 | -0.0113529 | -0.0057758 | -6.0279265 | 2.45E-09 | **** | post |
| PAUC43f_marine_benthic_group | -0.0096535 | 0.00142078 | -0.012442 | -0.0068649 | -6.7944837 | 2.01E-11 | **** | post |
| Desulfosarcinaceae | -0.0110396 | 0.00142078 | -0.0138281 | -0.0082511 | -7.7701018 | 2.19E-14 | **** | post |
| Sandaracinaceae | -0.0117327 | 0.00142078 | -0.0145212 | -0.0089441 | -8.2579109 | 5.44E-16 | **** | post |
| Thermoanaerobaculaceae | -0.0137129 | 0.00142078 | -0.0165014 | -0.0109243 | -9.6516511 | 5.14E-21 | **** | post |
| NB1-j | -0.0197525 | 0.00142078 | -0.022541 | -0.0169639 | -13.902559 | 7.49E-40 | **** | post |
| Comamonadaceae | -0.0297525 | 0.00142078 | -0.032541 | -0.0269639 | -20.940947 | 2.91E-79 | **** | post |
| Nocardiaceae | -0.0336139 | 0.00142078 | -0.0364024 | -0.0308253 | -23.658741 | 4.49E-96 | **** | post |
| Syntrophobacterales | -0.0348515 | 0.00142078 | -0.03764 | -0.032063 | -24.529828 | 1.45E-101 | **** | post |
| SG8-4 | 0.04534653 | 0.00174034 | 0.04193081 | 0.04876226 | 26.0561536 | 2.87E-111 | **** | industrial |
| Woesearchaeales | 0.02742574 | 0.00174034 | 0.02401002 | 0.03084146 | 15.7588527 | 1.91E-49 | **** | industrial |
| Omnitrophaceae | 0.02633663 | 0.00174034 | 0.02292091 | 0.02975235 | 15.1330499 | 3.92E-46 | **** | industrial |
| Marine_Benthic_Group_D_and_DHVEG-1 | 0.0150495 | 0.00174034 | 0.01163378 | 0.01846523 | 8.6474571 | 2.48E-17 | **** | industrial |
| Dehalococcoidia | 0.01346535 | 0.00174034 | 0.01004963 | 0.01688107 | 7.73719846 | 2.79E-14 | **** | industrial |
| Anaerolineaceae | 0.01188119 | 0.00174034 | 0.00846547 | 0.01529691 | 6.82693981 | 1.62E-11 | **** | industrial |
| SCGC_AAA011-D5 | 0.01064356 | 0.00174034 | 0.00722784 | 0.01405929 | 6.11580025 | 1.45E-09 | **** | industrial |
| Bacteria (unclassified) | 0.00831683 | 0.00174034 | 0.00490111 | 0.01173255 | 4.77885787 | 2.07E-06 | **** | industrial |
| Sh765B-AG-111 | 0.0080198 | 0.00174034 | 0.00460408 | 0.01143552 | 4.60818437 | 4.67E-06 | **** | industrial |
| Thermoplasmata | 0.00777228 | 0.00174034 | 0.00435656 | 0.011188 | 4.46595646 | 9.01E-06 | **** | industrial |
| Thermoanaerobaculaceae | -0.0116832 | 0.00174034 | -0.0150989 | -0.0082674 | -6.7131575 | 3.42E-11 | **** | industrial |
| Desulfobulbaceae | -0.0125248 | 0.00174034 | -0.0159405 | -0.009109 | -7.1967324 | 1.33E-12 | **** | industrial |
| Woeseiaceae | -0.0149505 | 0.00174034 | -0.0183662 | -0.0115348 | -8.5905659 | 3.93E-17 | **** | industrial |
| Fusobacteriaceae | -0.015 | 0.00174034 | -0.0184157 | -0.0115843 | -8.6190115 | 3.12E-17 | **** | industrial |
| Nitrosopumilaceae | -0.0160396 | 0.00174034 | -0.0194553 | -0.0126239 | -9.2163687 | 2.23E-19 | **** | industrial |
| Desulfatiglandaceae | -0.024802 | 0.00174034 | -0.0282177 | -0.0213863 | -14.251237 | 1.34E-41 | **** | industrial |
| Comamonadaceae | -0.0267822 | 0.00174034 | -0.0301979 | -0.0233665 | -15.38906 | 1.77E-47 | **** | industrial |
| Syntrophobacterales | -0.0296535 | 0.00174034 | -0.0330692 | -0.0262377 | -17.038904 | 1.91E-56 | **** | industrial |
| Nocardiaceae | -0.0319307 | 0.00174034 | -0.0353464 | -0.028515 | -18.347401 | 7.11E-64 | **** | industrial |
| Desulfosarcinaceae | -0.0406436 | 0.00174034 | -0.0440593 | -0.0372278 | -23.353823 | 3.67E-94 | **** | industrial |
| Comamonadaceae | -0.0789109 | 0.00227039 | -0.0833669 | -0.0744549 | -34.756547 | 4.86E-167 | **** | pre |
| Nocardiaceae | -0.0632673 | 0.00227039 | -0.0677234 | -0.0588113 | -27.86629 | 7.27E-123 | **** | pre |
| Desulfatiglandaceae | -0.0226733 | 0.00227039 | -0.0271293 | -0.0182172 | -9.986511 | 2.58E-22 | **** | pre |
| SG8-4 | -0.0159406 | 0.00227039 | -0.0203966 | -0.0114846 | -7.0210841 | 4.41E-12 | **** | pre |
| Sva0485 | -0.0137129 | 0.00227039 | -0.0181689 | -0.0092568 | -6.0398767 | 2.28E-09 | **** | pre |
| JS1 | -0.0111881 | 0.00227039 | -0.0156442 | -0.0067321 | -4.9278417 | 9.94E-07 | **** | pre |
| Dehalococcoidia | -0.0066832 | 0.00227039 | -0.0111392 | -0.0022271 | -2.9436222 | 0.00332963 | ** | pre |
| Lokiarchaeia | -0.0056931 | 0.00227039 | -0.0101491 | -0.001237 | -2.50753 | 0.01233775 | * | pre |
| FW22 | -0.0052475 | 0.00227039 | -0.0097036 | -0.0007915 | -2.3112886 | 0.02104823 | * | pre |
| Latescibacteraceae | 0.00450495 | 0.00227039 | 4.89E-05 | 0.00896099 | 1.98421943 | 0.04754307 | * | pre |
| CK-2C2-2 | 0.0060396 | 0.00227039 | 0.00158357 | 0.01049564 | 2.66016231 | 0.00795305 | ** | pre |
| Calditrichaceae | 0.00643564 | 0.00227039 | 0.00197961 | 0.01089168 | 2.83459918 | 0.00469387 | ** | pre |
| Omnitrophaceae | 0.00717822 | 0.00227039 | 0.00272218 | 0.01163426 | 3.16166832 | 0.00162264 | ** | pre |
| Zixibacteria | 0.00757426 | 0.00227039 | 0.00311822 | 0.0120303 | 3.33610519 | 0.00088535 | *** | pre |
| Bacteroidetes_BD2-2 | 0.00757426 | 0.00227039 | 0.00311822 | 0.0120303 | 3.33610519 | 0.00088535 | *** | pre |
| Desulfosarcinaceae | 0.00772277 | 0.00227039 | 0.00326673 | 0.01217881 | 3.40151902 | 0.00070046 | *** | pre |
| Woesearchaeales | 0.0080198 | 0.00227039 | 0.00356376 | 0.01247584 | 3.53234667 | 0.0004334 | *** | pre |
| NB1-j | 0.00826733 | 0.00227039 | 0.00381129 | 0.01272336 | 3.64136972 | 0.0002871 | *** | pre |
| Anaerolineaceae | 0.01113861 | 0.00227039 | 0.00668258 | 0.01559465 | 4.90603704 | 1.11E-06 | **** | pre |
| Marine_Benthic_Group_D_and_DHVEG-1 | 0.0149505 | 0.00227039 | 0.01049446 | 0.01940653 | 6.58499194 | 7.83E-11 | **** | pre |
